# Perplexing paradoxical reactions: navigating the complexity of protracted tuberculosis meningitis—a case report

**DOI:** 10.3389/fimmu.2024.1441945

**Published:** 2024-10-31

**Authors:** Megan S. Gooding, Dima A. Hammoud, Brian Epling, Joseph Rocco, Elizabeth Laidlaw, Safia Kuriakose, Mansi Chaturvedi, Frances Galindo, Stella V. Ma, Harry Mystakelis, April Poole, Kelly Russo, Maunank Shah, Joseph L. Malone, Adam W. Rupert, Irini Sereti, Maura Manion

**Affiliations:** ^1^ Clinical Research Directorate, Frederick National Laboratory for Cancer Research, Frederick, MD, United States; ^2^ Center for Infectious Disease Imaging, NIH Clinical Center, NIH, Bethesda, MD, United States; ^3^ National Institute of Allergy and Infectious Diseases, National Institutes of Health, Bethesda, MD, United States; ^4^ Division of Pulmonary and Critical Care Medicine, University of Maryland, Baltimore, Baltimore, MD, United States; ^5^ Division of Pulmonary Medicine, National Heart, Lung, and Blood Institutes, National Institutes of Health, Bethesda, MD, United States; ^6^ Howard County Department of Health, Columbia, MD, United States; ^7^ Department of Health, Baltimore City TB Program, Baltimore, MD, United States; ^8^ Montgomery County Department of Health, Silver Spring, MD, United States; ^9^ AIDS Monitoring Laboratory, Frederick National Laboratory for Cancer Research, Frederick, MD, United States

**Keywords:** tuberculous meningitis, paradoxical reaction, case series, immunocompetent, therapeutic drug monitoring

## Abstract

Tuberculous meningitis (TBM) has considerable mortality and morbidity, and it often presents therapeutic challenges when complicated by paradoxical reactions (PRs). Here, the clinical course of four cases of TBM patients complicated by PRs in a longitudinal TB cohort is described while also providing insights from the larger clinical cohort. Research flow cytometry, biomarker analysis, and drug concentrations were performed on available samples. All participants were initiated on standard antituberculosis therapy (ATT) and enrolled at the onset of PRs (PR group) or 2–4 months after the start of ATT (controls). The four TBM participants highlighted here presented with fevers, headaches, neurological deficits, and fatigue at the initial presentation. Upon diagnosis, all were initiated on rifampin, isoniazid, pyrazinamide, and ethambutol (RHZE) at standard doses and on corticosteroids. The median time to first PR was 37 days with recrudescence of initial TBM signs and symptoms at the time of PR. At the time of referral, all participants had low drug concentrations requiring dose optimization and regimen intensification as well as recrudescent flares upon corticosteroid taper, with one individual developing enlargement of tuberculoma 1 year following completion of ATT. Based on biomarkers and flow cytometry, PRs are characterized by elevated interferon-gamma and ferritin levels in the plasma compared to controls. In the TBM participants, T-cell activation with elevated levels of inflammatory biomarkers in the cerebrospinal fluid (CSF) was seen at the time of PR. These unique and highly detailed TBM cases provide insights into the pathogenesis of PRs, which may assist with future diagnostics and treatment.

## 1 Introduction

Tuberculosis (TB) has a significant global burden, with 10 million people developing the disease in 2022, accounting for an estimated 1.3 million deaths (1), making TB the second leading cause of death from infectious diseases after COVID-19 (1). Paradoxical reactions (PRs) are a longstanding observed complication of TB, likely a consequence of dysregulated immune responses. A PR is a worsening of a patient’s pre-existing clinical and/or radiological disease or the appearance of a new disease after starting antituberculosis therapy (ATT). This is a distinct yet similar immunologic phenomenon as immune reconstitution inflammatory syndrome (IRIS) in patients with HIV, which occurs following antiretroviral therapy initiation (2–9).

PRs and IRIS are diagnoses of exclusion; however, in IRIS, the initiation of antiretroviral therapy with subsequent HIV virologic response is a helpful clue to providers that they are managing a dysregulated immune response (7, 10, 11). In contrast, PRs can be challenging to distinguish from treatment failure given the length of time needed to grow TB and the difficulty of culturing TB from extrapulmonary sites. PRs can occur in 3%–25% of patients without HIV starting ATT (4, 5, 12–14). Comparable mechanisms to IRIS may be at play including an initial state of immune dysfunction due to TB itself, a large burden of mycobacterial disease, and restoration of immunity upon starting ATT (4, 11, 13, 15–18). The pathogenesis of PRs in people without HIV is still poorly understood and needs further characterization.

PR within TB meningitis (TBM) is particularly concerning, as it may lead to further neurological complications and mortality (10–13, 15, 16, 19). TBM and intracranial tuberculomas complicate an estimated 3.67% and 0.52% of TB disease cases, respectively (18), and are especially dangerous, with estimated mortality from 20% to over 40% in some cohorts (9, 20–23).

Here, we share our experience of four TBM cases complicated by PRs and recrudescent flares requiring prolonged treatment with ATT and corticosteroids from a larger clinical cohort investigating PRs in patients without HIV. Data from the entire cohort including participants without PRs (controls) and those with PRs (PRs) are presented alongside an in-depth analysis of four TBM cases in the PR group. Therapeutic drug monitoring (TDM) via serum was conducted on all participants. Research on cerebrospinal fluid (CSF) drug concentrations, systemic and local (CSF) inflammatory biomarkers, and flow cytometry were performed to further delineate and characterize PRs in TBM.

## 2 Methods

The participants were enrolled in an observational study to characterize PRs in patients with TB and without HIV at the National Institutes of Health (NIH) on an institutional review board-approved protocol (NCT04052022). All participants provided written informed consent prior to study enrollment. To qualify as a PR, participants had to have microbiologically confirmed TB and at least two signs or symptoms of a PR including fever, recrudescence of TB presentation, or appearance of new sequelae of TB with concordant findings on exam. Furthermore, these signs/symptoms could not be explained by a newly acquired infection, side effects of ATT, the presence of drug resistance, or any other condition. People with active TB without PRs were enrolled in the control group after 2–4 months of ATT therapy to match average PR presentation timing (3, 10, 13, 16). Demographic data are reported in **Supplementary Table 2**. Detailed methods are included in the **Supplementary Material**.

## 3 TBM participant descriptions

**Figure 1:**
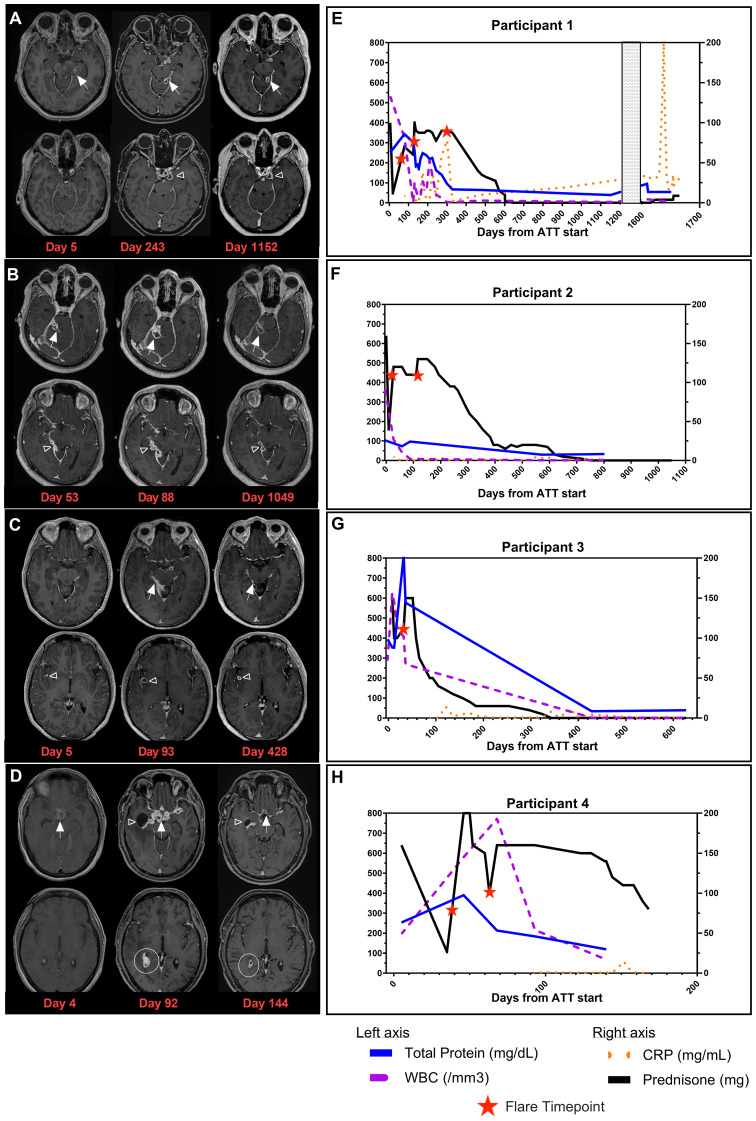
Clinical images and timelines for TBM participants. Post contrast-enhanced T1-weighted images at two levels in the brain for each participant. **(A)** Brain MRI of Participant 1 at D5 (time of initial diagnosis), D243 (during recrudescent PRs), and D1152 (following resolution of PRs). Loculated leptomeningeal thickening and enhancement in the left perimesencephalic (white arrows) and suprasellar (open arrowhead) cisterns. **(B)** Brain MRI of Participant 2 at D53 (time of PR referral), D88 (time of recrudescent PRs), and D1049 (resolution of PRs). Multiloculated leptomeningeal rim-enhancing lesions are seen in the right perimesencephalic cistern (white arrows) and along the inferior aspect of the right tentorium cerebelli (open arrowhead). **(C)** Brain MRI of Participant 3 at D5 (time of initial diagnosis), D93 (time of PR referral), and D428 (resolution of PRs). Leptomeningeal thickening and enhancement in the right perimesencephalic cistern (white arrows) with a rim-enhancing lesion in the right sylvian fissure (open arrowhead). **(D)** Brain MRI of Participant 4 at D4 (time of initial diagnosis), D92 (time of PR referral), and D144 (while receiving treatment for PRs). Thick necrotic enhancement in the suprasellar region (white arrows) with extension along the course of the major vessels, right lateral ventricular ependymal and choroid plexus enhancement (white circle), and entrapment of the right temporal horn (open arrowhead). **(E)** Timeline for Participant 1. **(F)** Timeline for Participant 2. **(G)** Timeline for Participant 3. **(H)** Timeline for Participant 4. Timeline representation of CSF profile (WBC and total protein) in addition to CRP (plasma) and prednisone dose plotted over time. X-axis is denoted as time from the start of ATT for all timelines, and length varies due to the unique clinical course for each participant. Total protein (mg/dL) and WBC (/mm^3^) from CSF findings are plotted on the left y-axis. Prednisone (mg) and CRP (mg/mL) are plotted on the right y-axis. Patients on dexamethasone or other corticosteroids had doses converted to prednisone for presentation on timeline. Red stars represent flare timepoints and need for increase of steroids to manage symptoms. TBM, tuberculous meningitis; PRs, paradoxical reactions; CSF, cerebrospinal fluid; WBC, white blood cell; CRP, C-reactive protein; ATT, antituberculosis therapy.

Full demographics, medication doses, and relevant clinical information are provided in **Supplementary Tables 1–5**. **Figure 1** provides timelines of participants’ clinical courses.

Participant 1, a 39-year-old woman, was admitted with worsening headaches and right-sided weakness; imaging demonstrated a lobulated rim-enhancing left thalamic lesion with vasogenic edema and mass effect. She had two inconclusive brain biopsies. One month later, she was readmitted with fevers. Her QuantiFERON Gold was positive, and lumbar puncture (LP) was performed. CSF cultures eventually grew TB. She initiated rifampin (RIF), isoniazid (INH), pyrazinamide (PZA), and ethambutol (EMB) (RHZE) and dexamethasone with moxifloxacin (MOX) added to her regimen at Day 50. Ninety-three days after starting ATT and corticosteroid taper, she was referred to the NIH for new visual changes and tinnitus alongside continued right-sided weakness. While imaging showed a decreased size of the thalamic lesion, there was a worsening of leptomeningeal enhancement, which was concerning for PR. LP was performed with negative TB PCR and acid-fast bacillus (AFB) culture. Two-hour serum ATT drug levels revealed RIF, INH, and EMB levels below the reference range, and she was a rapid acetylator of INH. INH and RIF doses were increased, EMB was stopped, linezolid (LNZ) was started, and corticosteroids were increased. On Day 118, she had a recrudescent PR with nausea, vomiting, and headaches following prednisone taper, requiring an increased dose of prednisone. During corticosteroid taper, she had recrudescent flares characterized by fever, headaches, or a decrease in vision and worsening brain MRI findings (**Figure 1A**, Day 243). She stopped corticosteroids 643 days and ATT 1,160 days after initiation (**Figure 1E**), with clinical improvement to her baseline health and radiological improvement (**Figure 1A**, Day 1152). She represented approximately 500 days after ATT cessation with fatigue, polydipsia, and polyuria. Her brain MRI showed an increased bulk of enhancing suprasellar lesions and compression of the pituitary stalk. This caused panhypopituitarism requiring hydrocortisone and desmopressin therapy. FDG-PET scan was notable for increased metabolism in the suprasellar enhancing tissues and one axillary lymph node (LN). She had two negative TB PCRs in the CSF, and an axillary LN biopsy showed follicular hyperplasia with negative AFB stains. Neurosurgery deemed biopsy of prior tuberculoma to be of considerable risk, and therefore, ATT was re-started while awaiting culture results. Imaging showed a decrease in the size of the tuberculoma immediately prior to ATT resumption. CSF and LN cultures were finalized as no growth, and CSF metagenomics returned as negative at the time of manuscript submission, suggesting that the presentation was consistent with a PR, and the decision was made to stop ATT and monitor her off therapy.

Participant 2, a 34-year-old man, presented with left-sided weakness, facial droop, fevers, headaches, and pleural effusion. He was diagnosed with TB with positive GeneXpert on pleural fluid and lymphocytic predominant CSF on LP, which eventually grew TB. He was started on ATT with RHZE with MOX and prednisolone. Twenty-eight days later, he was readmitted with worsening headaches, double vision, neck pain, fever, and chills, with a brain MRI revealing right perimesencephalic leptomeningeal enhancement with adjacent parenchymal edema. The clinical presentation was consistent with a PR, and prednisone was increased. He was referred to the NIH on Day 44, and a brain MRI upon enrollment showed multiloculated leptomeningeal enhancing abnormalities in the right perimesencephalic cistern and along the inferior aspect of the right tentorium cerebelli (**Figure 1B**, Day 53) with adjacent brainstem and right cerebellar edema, despite adherence to all ATT medications and continuation on the same corticosteroid dose. LP was performed with a negative mycobacterial culture and TB PCR. Serum drug concentrations were below reference ranges for RIF, EMB, and INH, and his doses were increased. On Day 88 of ATT and during prednisone taper, he developed right-sided cranial nerve VII palsy and headaches. Imaging revealed interval worsening of the posterior fossa enhancing abnormalities (**Figure 1B**, Day 88) and increased edema. ATT was intensified with the addition of LNZ, and prednisone was increased. With these changes, he had a gradual resolution of his symptoms and decreased size of the enhancing abnormalities on MRI (**Figure 1B**, Day 1049). Similar to Participant 1, he developed recrudescent flares with headaches and visual changes throughout the corticosteroid taper. He completed corticosteroids 716 days after the initial onset of symptoms; however, they were resumed 120 days later due to recrudescence of PR symptoms with headaches and blurry vision but were quickly tapered (**Figure 1F**). His entire course of ATT lasted 951 days. He has remained asymptomatic for 1 year after ATT cessation.

Participant 3, a 31-year-old man, presented with fever, headaches, right-sided weakness, and altered mental status. Brain MRI revealed multiple enhancing lesions in the basal cisterns and right sylvian fissure (**Figure 1C**, Day 5) with surrounding vasogenic edema, and CSF TB PCR was positive. He was started on RHZE and dexamethasone. He was readmitted 1 week later due to fatigue and was found to have elevated transaminases, and RIF and INH were discontinued. Levofloxacin (LEVO) and LNZ were started with dexamethasone taper resumed when he was discharged the following day. On Day 32, he was readmitted with worsening headaches, seizures, and altered mental status. Brain MRI showed worsening of the nodular leptomeningeal enhancement in the basilar cisterns/right sylvian fissure and along cranial nerves with new bilateral frontal and cerebellar enhancing foci. His LP showed increased intracranial pressure with an opening pressure of 57 cm H_2_O, negative TB PCR, and negative mycobacterial culture. ATT was changed to RIF, LNZ, LEVO, and ETH. Due to concern for PRs, dexamethasone was increased (**Figure 1G**). His course was complicated by seizures, and he was placed on levetiracetam along with aspirin for stroke prevention. He improved following these interventions and was referred to the NIH where he presented on Day 92 of ATT. Two-hour serum drug levels of INH and EMB were found to be below reference ranges with delayed absorption of RIF, and doses were increased. Dexamethasone was switched to prednisone, and corticosteroid taper was started with recrudescence of headaches throughout the taper. Over the course of the next year, he had progressive improvement of symptoms, and subsequent brain MRIs showed a decreased extent of the leptomeningeal enhancement (**Figure 1C**, Day 428). He has remained asymptomatic for 18 months after treatment.

Participant 4, a 45-year-old man, admitted with malaise, fatigue, fevers, and encephalopathy and was found to have bilateral upper lobe cavitary lesions in the lungs. Sputum AFB smear and TB PCR were positive. Brain MRI demonstrated diffuse basal leptomeningeal enhancement with interpeduncular and suprasellar extension (**Figure 1D**, Day 4). LP revealed pleocytosis and elevated adenosine deaminase. He initiated RHZE along with dexamethasone. His clinical status improved with treatment initiation, and he was discharged. However, he was readmitted on Day 41 of ATT with blurry vision and visual hallucinations following the dexamethasone taper (**Figure 1H**). EMB was changed to LEVO, and dexamethasone was increased. MRI demonstrated marked worsening with the development of thick necrotic enhancement in the same distribution with extension along the course of the major vessels bilaterally. There was also right lateral ventricular ependymal and choroid plexus enhancement, entrapment of the right occipital horn, and transependymal CSF seepage. He was referred to the NIH for concern of PR. Brain MRI at the NIH demonstrated slight improvement of suprasellar disease but worsened ependymitis, choroiditis, adjacent edema, and right temporal horn entrapment (**Figure 1D**, Day 92). Two-hour serum drug levels of RIF and INH were below reference ranges, prompting increased doses and the addition of LNZ to his regimen. Dexamethasone were switched to an equivalent dose of prednisone. A repeat MRI of the brain showed improvement in all intracranial abnormalities (**Figure 1D**, Day 144). However, given the extent of enhancement on imaging, the recurrence of PR symptoms with corticosteroid taper, and the considerable morbidity associated with corticosteroids—including steroid-induced diabetes mellitus, weight gain, hypertension, and oral candidiasis—infliximab was administered. This was done to assist in corticosteroid taper once therapeutic ATT concentrations were achieved. He received one dose of infliximab and re-presented 11 days later with neutropenia (ANC of 0 per microliter), oral mucositis, and polymicrobial bacteremia with oral flora that was treated with cefepime. He was given three doses of granulocyte colony-stimulating factor with neutrophil recovery. No further doses of infliximab were given, and LNZ, prophylactic trimethoprim–sulfamethoxazole, and semaglutide (first dose given 2 days prior to neutropenia) were also held. Corticosteroid taper was resumed with symptoms of recrudescent flares of visual hallucinations. Following the infliximab dose, his prednisone was tapered to half the initial dose at the time of manuscript submission.

## 4 Observations during the course of care

All four participants were initiated on RHZE at standard doses, vitamin B6, and corticosteroids at the time of TBM diagnosis (17, 18, 24, 25). The median time to initial PR was 37 days (**Supplementary Table 2**) with recrudescence of initial symptoms requiring hospitalization with new or worsening findings on MRI. Mycobacterial cultures, molecular testing, and other microbiologic workup from CSF at the time of PR referral were negative.

**Figure 2:**
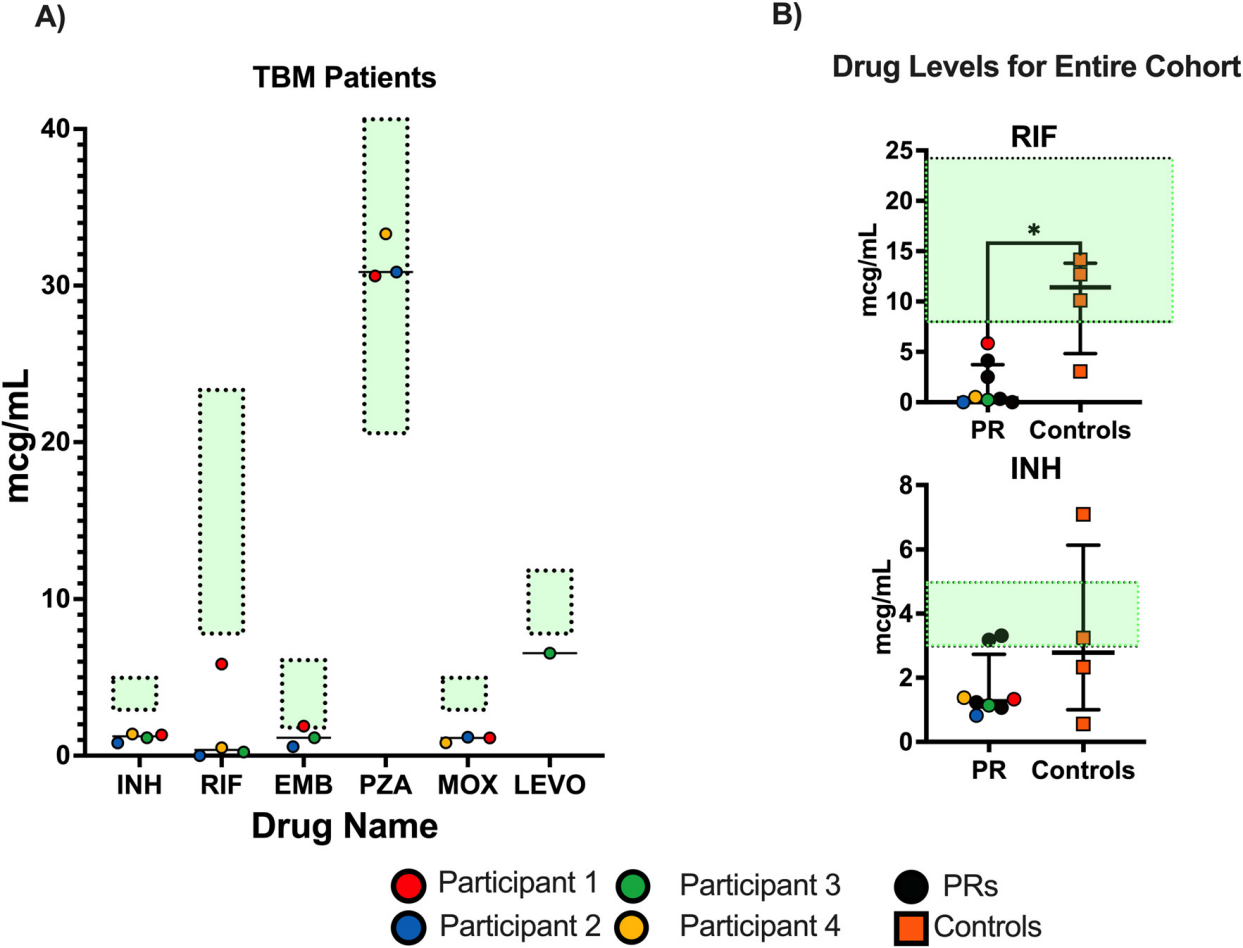
Peak serum antituberculosis therapy drug concentrations upon enrollment to NIH protocol. Reference ranges as reported by the University of Florida Infectious Disease Pharmacokinetic Laboratory are denoted as green zones in both panels. Drug concentrations were performed at a 2-hour timepoint in serum. All patients were evaluated for delayed absorption. **(A)** TBM participant drug concentrations for all ATT drugs at time of enrollment. Case 1 is red, Case 2 is blue, Case 3 is green, and Case 4 is yellow. **(B)** Drug concentrations of all paradoxical reaction (PR) patients compared to TB controls enrolled in the larger cohort. Mann–Whitney t-tests were performed; significant values p<0.05 denoted as *. Only RIF and INH were reported, as most patients referred to as controls were in continuation phase of ATT. INH, isoniazid; PZA, pyrazinamide; EMB, ethambutol; RIF, rifampin; MOX, moxifloxacin; LEVO, levofloxacin; TBM, tuberculous meningitis; ATT, antituberculosis therapy.

Each participant presented to the NIH within 3 months of ATT initiation; corticosteroids were switched to prednisone for a more gradual corticosteroid taper. The decision of when to taper was guided by close follow-up of clinical symptoms, physical exam, laboratory findings including C-reactive protein and CSF profile, and imaging. At enrollment, Rifampin, Isoniazid, Ethambutol (RHE) serum peak concentrations were below reference ranges in all four participants (**Figure 2A**; **Supplementary Table 4**). These findings were consistent with our overall protocol cohort where we have found significantly lower concentrations of RIF in participants who developed PRs (**Figure 2B**) compared to controls.

All four participants required ATT intensification with RIF dosing of 15 mg/kg or higher, fluoroquinolone, and LNZ. In Participants 1–3, total therapy duration ranged from 9 to 32 months of corticosteroids and 14 to 40 months of ATT. Participant 4 remains on ATT and corticosteroids at the time of manuscript submission. In the setting of prolonged corticosteroids over 12 months, a dual-energy X-ray absorptiometry (DEXA) scan was obtained in Participants 1 and 2 with T scores of −1.1 and −1.9, respectively, consistent with low bone mineral density of the lumbar spine.

## 5 Research findings

**Figure 3:**
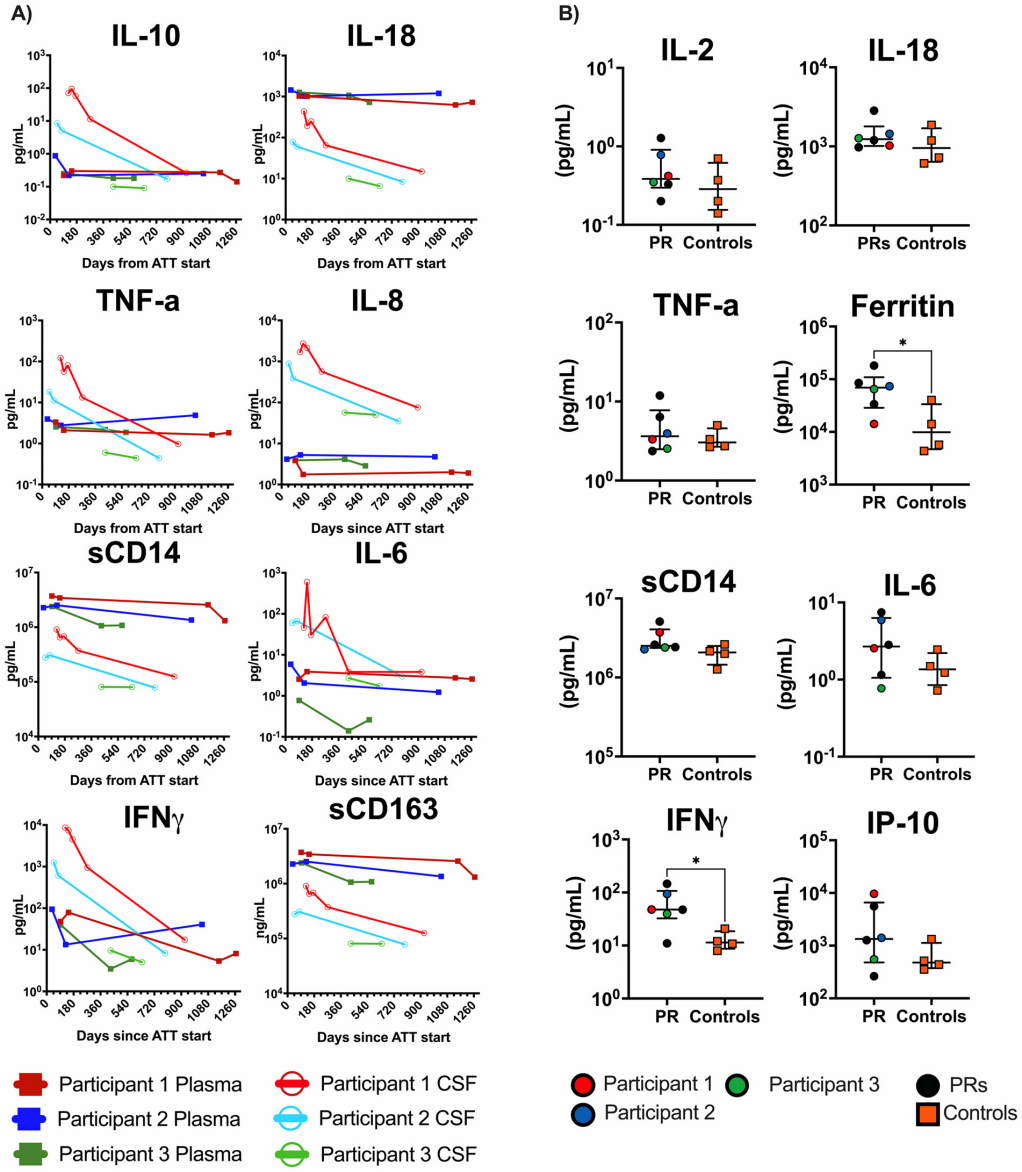
Plasma and CSF biomarker results from TBM participants and general cohort. **(A)** All open circles are CSF, and solid squares are plasma values. Case Participant 1 is red, Case Participant 2 is blue, and Case Participant 3 is green. Biomarker data are in pg/mL. X-axis is days from ATT start. **(B)** Baseline plasma values for all paradoxical reaction (PR) cases compared to controls (n = 4) enrolled in the larger cohort. Mann–Whitney t-tests were performed; significant values p<0.05 denoted as *. Cases are denoted in the PR group by circles and controls are denoted by orange squares. Case 4 was not included in either analysis. CSF, cerebrospinal fluid; TBM, tuberculous meningitis; ATT, antituberculosis therapy.

In the three TBM participants with research drug concentrations measured in the CSF, RIF penetration was low to undetectable (**Supplementary Table 6**). Myeloid (IL-6 and IL-8) and Th1 (IFN-γ, MIG, TNF-α, IP-10, and IL-10) soluble biomarkers were elevated at the time of recrudescent PRs in the CSF (**Figure 3A**; **Supplementary Figure 1C**) compared to follow-up timepoints. Notably, IL-18, sCD14, and sCD163, also suggestive of myeloid activation, were notably higher in the plasma compared to CSF (**Figure 3A**). In our larger cohort, we found significantly higher levels of both IFN-γ and ferritin in the PR group compared to controls in the plasma at the time of referral (**Figure 3B**).

**Figure 4:**
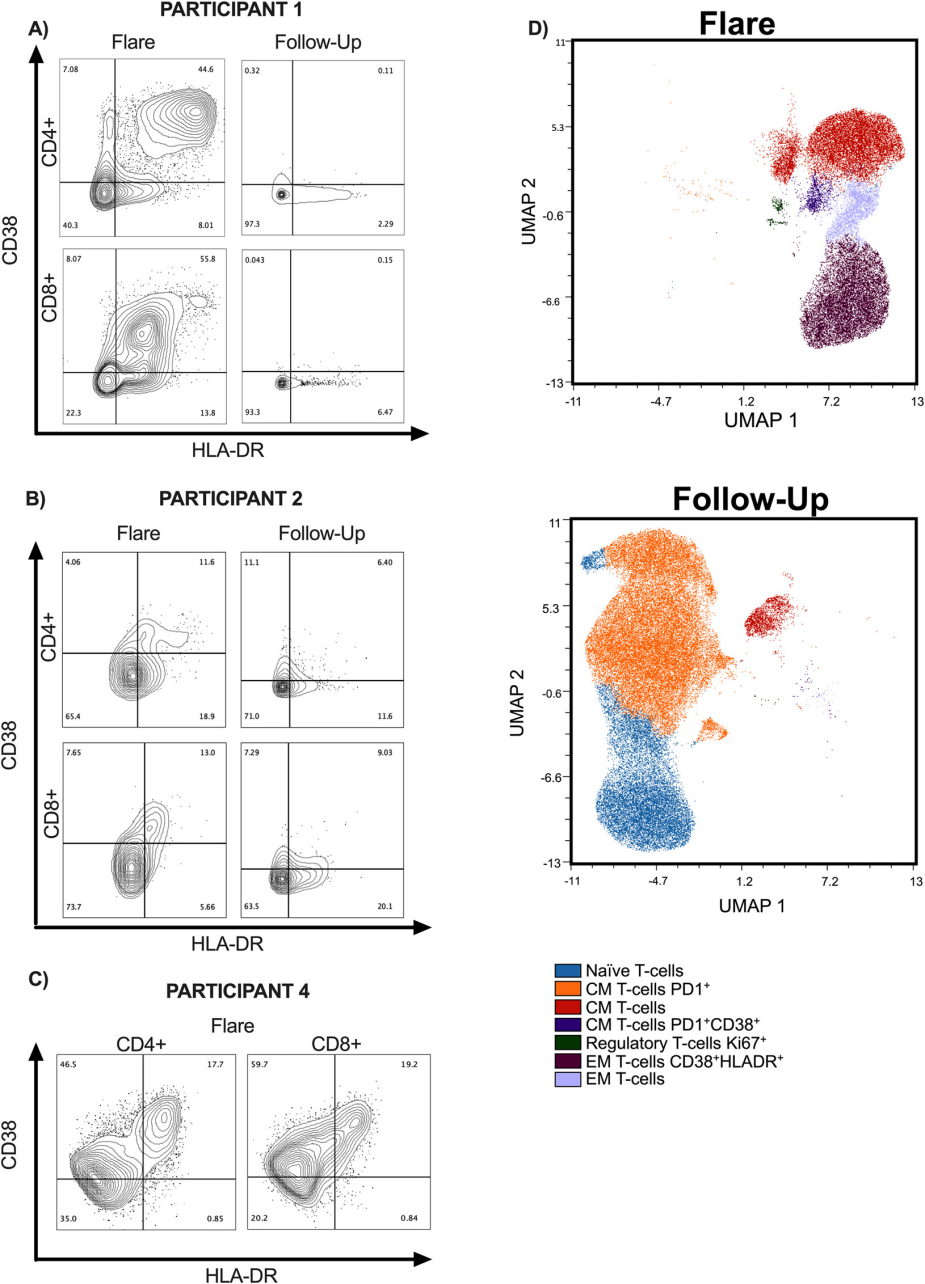
Flow cytometry data on TBM participants at time of recrudescent flare and follow-up. **(A–C)** Contour plots of CD4+ and CD8+ T cells in CSF and are presented at recrudescent flare and follow-up timepoints. For the follow-up time points, Participants 1 and 2 were on ATT with no active flare symptoms. Participant 1 had completed steroid taper in follow-up timepoint and Participant 2 was on 17.5 mg of prednisone every other day. Participant 4 only has the recrudescent flare timepoint, as he is still receiving treatment. PBMC data can be found in supplemental materials. **(D)** High dimensional analyses using FlowSom demonstrates the differences in CD4+ T-cell subsets in the CSF at acute and follow-up timepoints for Participants 1 and 2. TBM, tuberculous meningitis; CSF, cerebrospinal fluid; PBMCs, peripheral blood mononuclear cells; ATT, antituberculosis therapy.

In the CSF, there was a marked increase of activated T cells at the time of recrudescent PRs (**Figures 4A–D**). There is a distinct double-positive population of HLA-DR+CD38+ CD8+ and CD4+ T cells (**Figures 4A, B**) and a small Ki-67 regulatory CD4+ T-cell population (**Figure 4B**) at flare timepoints compared to recovery timepoints. In the peripheral blood, the activated population seen during the flare was substantially decreased, suggesting distinct compartmental mechanisms (**Supplementary Figure 3**).

Six-hour *in vitro* stimulations with PPD antigen were performed using CSF cells and peripheral blood mononuclear cells at the time of recrudescent PRs. A strong polyfunctional cytokine response to TB antigens from CD4+ T cells in the CSF and periphery was seen in Participants 1 and 4, while an attenuated CD4+ T-cell response was found in Participant 2 (**Supplementary Figure 2**).

## 6 Discussion

Here, we described four cases of TBM complicated by PRs in the backdrop of our clinical cohort studying the pathogenesis of PRs. Recrudescence of PR symptoms with corticosteroid taper was noted in all four participants, requiring prolonged treatment with ATT and corticosteroids. Participant 1 even had a PR with enlargement of a tuberculoma 1 year following therapy, which has been observed in the literature with one case reported 5 years following ATT (26). The side effects of corticosteroids were notable, with two participants developing osteopenia, and Participant 4 developed uncontrolled diabetes mellitus, hypertension, weight gain, and oral candidiasis.

Serum drug concentrations below reference ranges were observed in our cohort and may be a risk factor for PRs in TB. It is feasible that low drug concentrations may lead to persistent antigen burden, triggering subsequent inflammatory reactions. TDM and adjusting doses to achieve reference ranges, along with the utilization of high-dose RIF and intensification agents, may reduce antigen burden, limiting further morbidity and mortality. Trials are ongoing to address concerns, including overall outcomes and poor CSF drug penetration, regarding the use of traditional TB regimens for TBM (9, 27). TDM may be a useful tool to optimize TBM regimens, and TDM should be obtained at the time of PR.

However, despite reaching target serum drug levels, symptoms of PRs persisted in each TBM participant, suggesting that other factors contribute to the development of PRs. Inflammatory biomarkers and flow cytometry provided insight into the pathogenesis of PRs. Notably, IFN-γ and ferritin were elevated at the time of PR in the plasma of our cohort and may be possible biomarkers of interest for PR diagnosis (28–30). Increased activation of CD4+ T cells and polyfunctional responses targeting TB antigens that were restricted to the CSF were observed during PRs in our TBM participants. The influx of activated antigen-specific T cells and Th1/myeloid-origin inflammatory or regulatory mediators in the CSF at the time of PR is reminiscent of studies on mycobacterial IRIS related to HIV (11, 31, 32). Additionally, distinct differences in biomarker and flow cytometry results were observed between CSF and blood samples, indicating the need for both sample types to obtain a comprehensive understanding. The drivers of dysregulated immune response may be multifactorial including pre-existing lymphopenia in the setting of TB (4, 33), antigen burden or inappropriately treated antigen, and underlying host factors including genetics, metabolism, and microbiome (30, 34–38).

Further deciphering the pathogenesis of PRs in patients without HIV will be critical to inform the best approaches to diagnosis and management. PR evaluation is still dependent on microbiologic sampling alongside clinical context, and identifying distinct immunologic signatures for PRs compared to treatment failure will aid clinicians in a timely diagnosis. Some patients may benefit from immune modulation in cases with persistent PRs, limiting the need for prolonged corticosteroid exposure (6, 27, 39–47). Understanding the key immunologic drivers in PRs will help clinicians choose the optimal immunomodulator for each case.

A limitation of our series is that with only a small number of samples analyzed with drug concentrations, flow cytometry, and biomarker analysis, our preliminary findings will need to be confirmed in a larger cohort. Moreover, all TBM participants received high-dose corticosteroids, likely diminishing immune responses and opening the possibility of disparate findings in people who can be studied at the initial PR presentation. In summary, we propose possible explanations for PR presentations by reviewing in-depth complicated PR participants from both the clinical and immunological perspectives and suggest possible pathways that could be amenable to novel therapeutic interventions beyond corticosteroids.

## 7 Patient perspective

Paradoxical reactions of TBM can lead to considerable comorbidity for patients including neurological complications and increased duration of corticosteroids and ATT. Clinicians should discuss and monitor adverse effects of corticosteroids and immune modulators with patients. For patients and their families, this diagnosis can be perplexing, daunting, and frustrating. Emphasizing close follow-up and communication of symptoms while tapering corticosteroids is important, as recrudescent flares may emerge.

## Data Availability

The raw data supporting the conclusions of this article will be made available by the authors, without undue reservation, upon request.
