# Supplementary material for: Perplexing paradoxical reactions: navigating the complexity of protracted tuberculosis meningitis—a case report
Source: Front Immunol. 2024 Oct 31;15:1441945. doi: 10.3389/fimmu.2024.1441945 (PMC11570994; doi:10.3389/fimmu.2024.1441945)
Supplement: Supplementary file 1 [file Presentation1.pptx]

## Slide 1
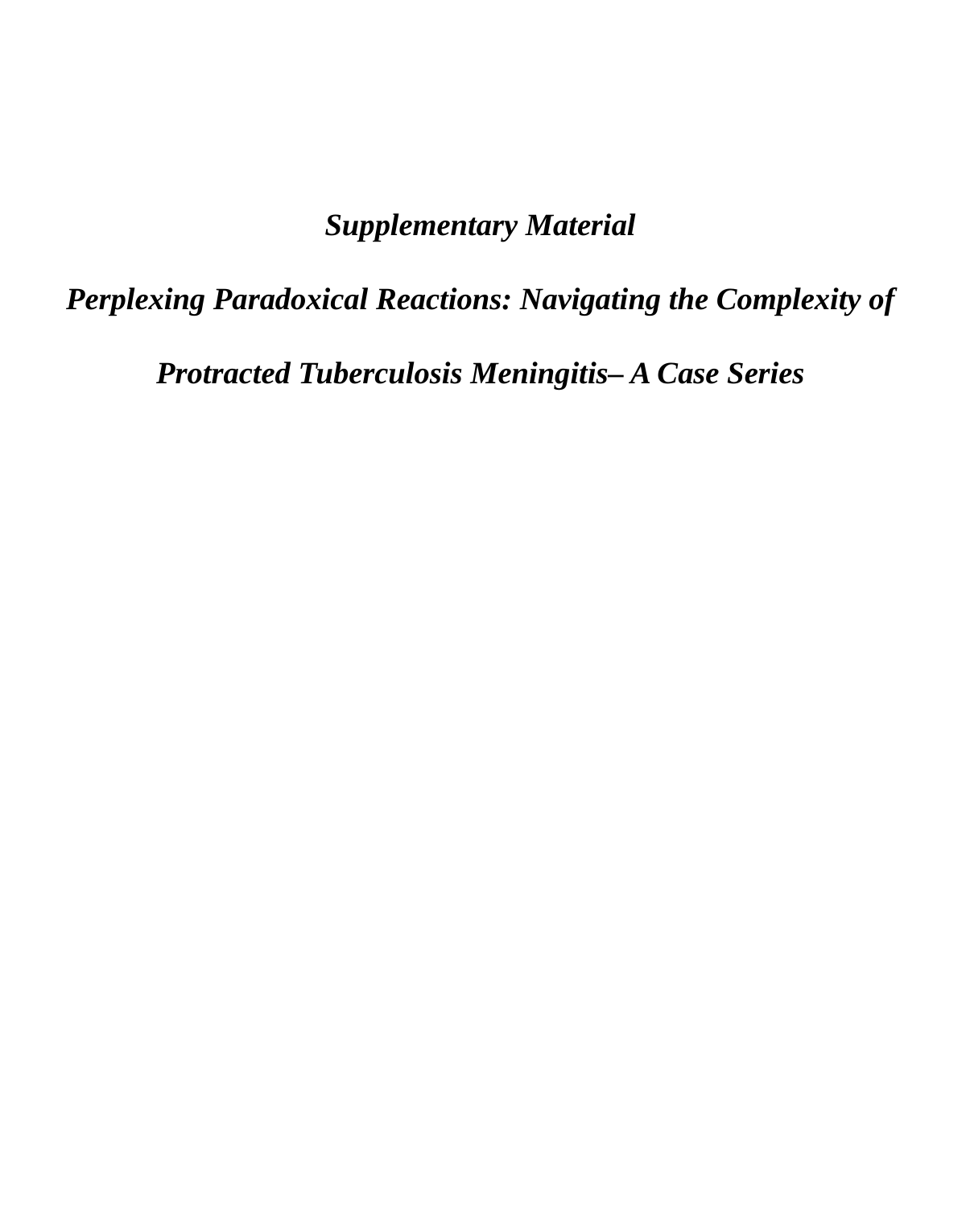

# Supplementary Material Perplexing Paradoxical Reactions: Navigating the Complexity of Protracted Tuberculosis Meningitis– A Case Series

## Slide 2
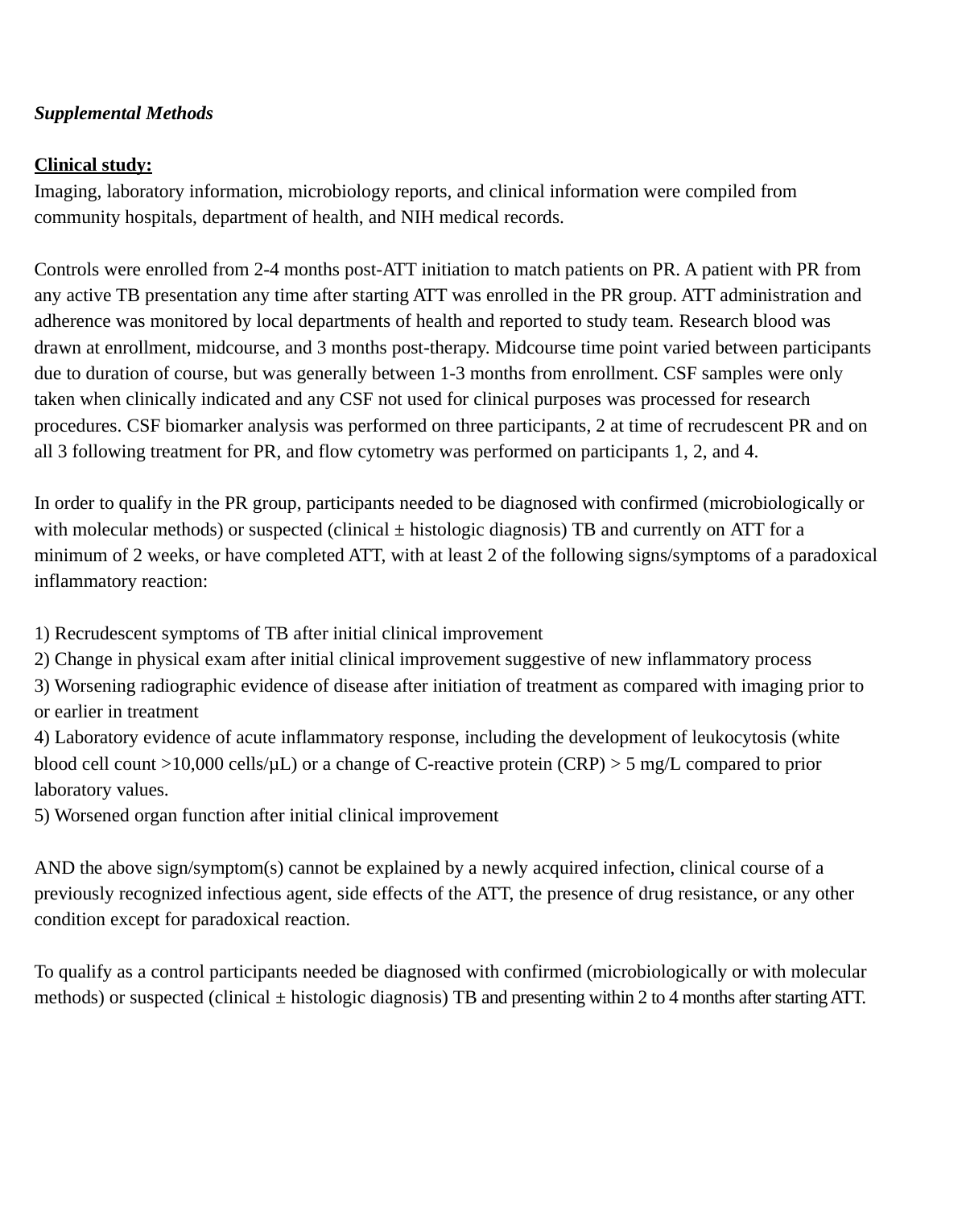

# Supplemental Methods Clinical study: Imaging, laboratory information, microbiology reports, and clinical information were compiled from community hospitals, department of health, and NIH medical records. Controls were enrolled from 2-4 months post-ATT initiation to match patients on PR. A patient with PR from any active TB presentation any time after starting ATT was enrolled in the PR group. ATT administration and adherence was monitored by local departments of health and reported to study team. Research blood was drawn at enrollment, midcourse, and 3 months post-therapy. Midcourse time point varied between participants due to duration of course, but was generally between 1-3 months from enrollment. CSF samples were only taken when clinically indicated and any CSF not used for clinical purposes was processed for research procedures. CSF biomarker analysis was performed on three participants, 2 at time of recrudescent PR and on all 3 following treatment for PR, and flow cytometry was performed on participants 1, 2, and 4. In order to qualify in the PR group, participants needed to be diagnosed with confirmed (microbiologically or with molecular methods) or suspected (clinical ± histologic diagnosis) TB and currently on ATT for a minimum of 2 weeks, or have completed ATT, with at least 2 of the following signs/symptoms of a paradoxical inflammatory reaction: 1) Recrudescent symptoms of TB after initial clinical improvement 2) Change in physical exam after initial clinical improvement suggestive of new inflammatory process 3) Worsening radiographic evidence of disease after initiation of treatment as compared with imaging prior to or earlier in treatment 4) Laboratory evidence of acute inflammatory response, including the development of leukocytosis (white blood cell count >10,000 cells/µL) or a change of C-reactive protein (CRP) > 5 mg/L compared to prior laboratory values. 5) Worsened organ function after initial clinical improvement AND the above sign/symptom(s) cannot be explained by a newly acquired infection, clinical course of a previously recognized infectious agent, side effects of the ATT, the presence of drug resistance, or any other condition except for paradoxical reaction. To qualify as a control participants needed be diagnosed with confirmed (microbiologically or with molecular methods) or suspected (clinical ± histologic diagnosis) TB and presenting within 2 to 4 months after starting ATT.

## Slide 3
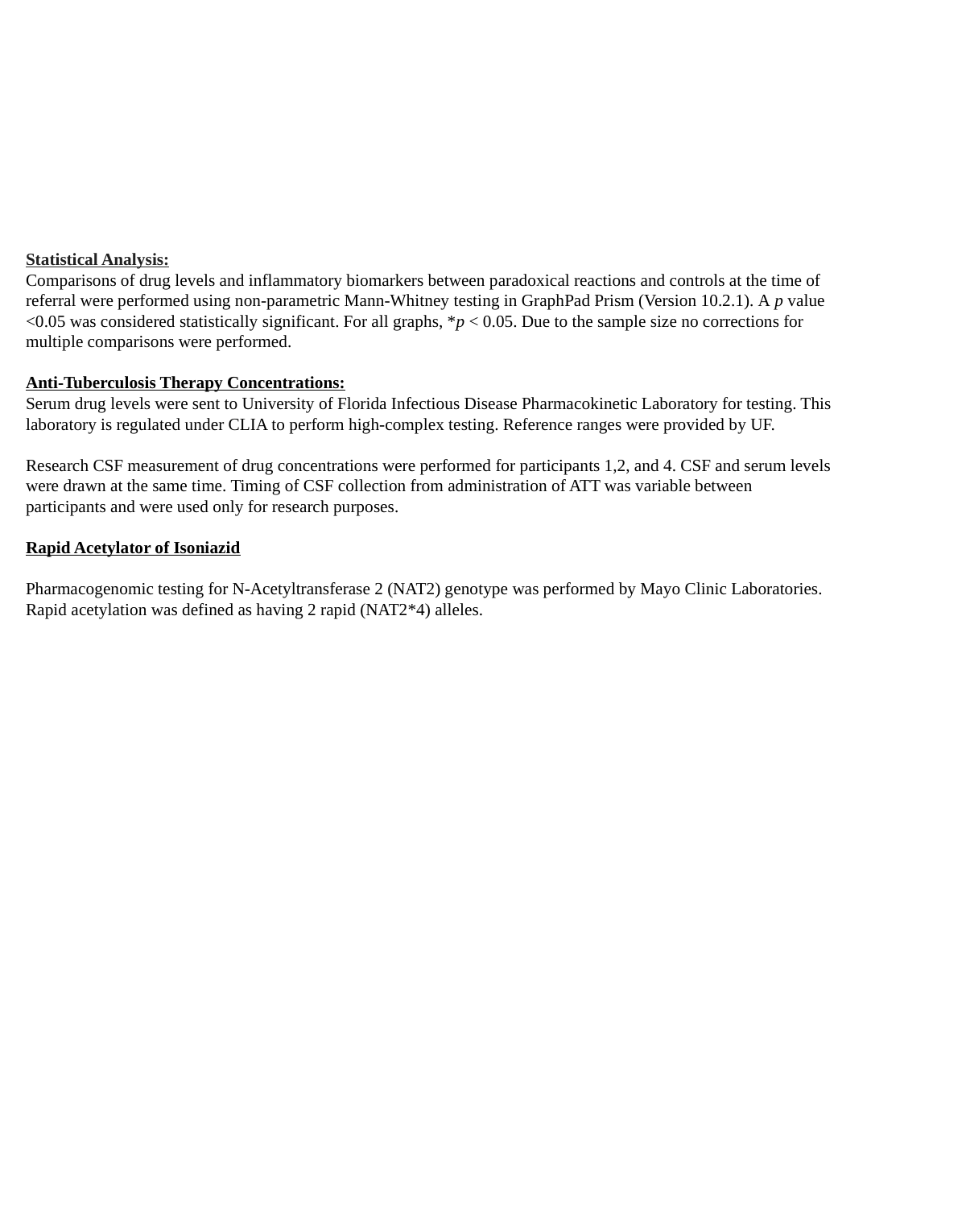

# Statistical Analysis: Comparisons of drug levels and inflammatory biomarkers between paradoxical reactions and controls at the time of referral were performed using non-parametric Mann-Whitney testing in GraphPad Prism (Version 10.2.1). A p value <0.05 was considered statistically significant. For all graphs, *p < 0.05. Due to the sample size no corrections for multiple comparisons were performed.  Anti-Tuberculosis Therapy Concentrations: Serum drug levels were sent to University of Florida Infectious Disease Pharmacokinetic Laboratory for testing. This laboratory is regulated under CLIA to perform high-complex testing. Reference ranges were provided by UF. Research CSF measurement of drug concentrations were performed for participants 1,2, and 4. CSF and serum levels were drawn at the same time. Timing of CSF collection from administration of ATT was variable between participants and were used only for research purposes. Rapid Acetylator of Isoniazid Pharmacogenomic testing for N-Acetyltransferase 2 (NAT2) genotype was performed by Mayo Clinic Laboratories. Rapid acetylation was defined as having 2 rapid (NAT2*4) alleles.

## Slide 4
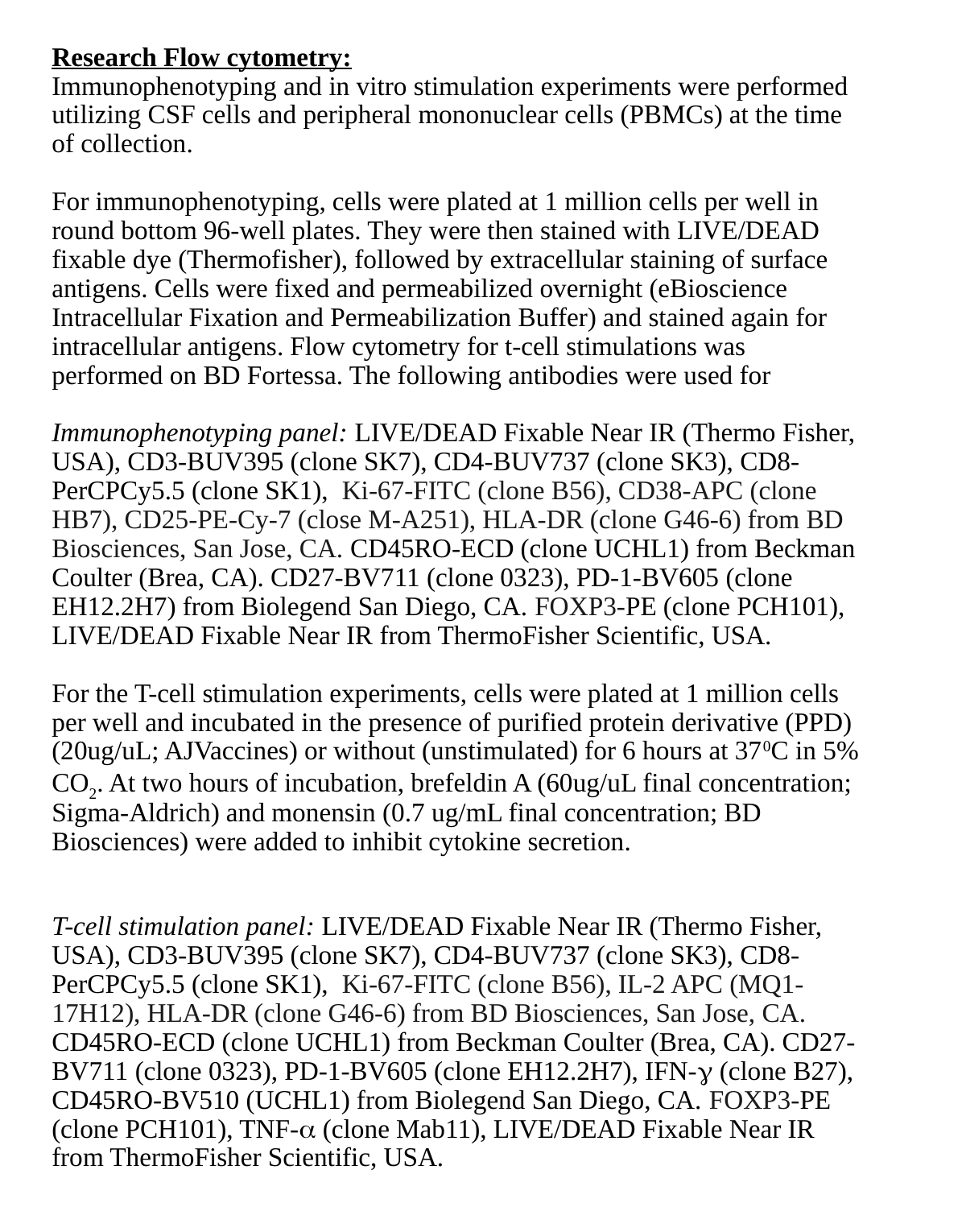

Research Flow cytometry:
Immunophenotyping and in vitro stimulation experiments were performed utilizing CSF cells and peripheral mononuclear cells (PBMCs) at the time of collection.
 
For immunophenotyping, cells were plated at 1 million cells per well in round bottom 96-well plates. They were then stained with LIVE/DEAD fixable dye (Thermofisher), followed by extracellular staining of surface antigens. Cells were fixed and permeabilized overnight (eBioscience Intracellular Fixation and Permeabilization Buffer) and stained again for intracellular antigens. Flow cytometry for t-cell stimulations was performed on BD Fortessa. The following antibodies were used for
 
Immunophenotyping panel: LIVE/DEAD Fixable Near IR (Thermo Fisher, USA), CD3-BUV395 (clone SK7), CD4-BUV737 (clone SK3), CD8-PerCPCy5.5 (clone SK1), Ki-67-FITC (clone B56), CD38-APC (clone HB7), CD25-PE-Cy-7 (close M-A251), HLA-DR (clone G46-6) from BD Biosciences, San Jose, CA. CD45RO-ECD (clone UCHL1) from Beckman Coulter (Brea, CA). CD27-BV711 (clone 0323), PD-1-BV605 (clone EH12.2H7) from Biolegend San Diego, CA. FOXP3-PE (clone PCH101), LIVE/DEAD Fixable Near IR from ThermoFisher Scientific, USA.
 
For the T-cell stimulation experiments, cells were plated at 1 million cells per well and incubated in the presence of purified protein derivative (PPD) (20ug/uL; AJVaccines) or without (unstimulated) for 6 hours at 370C in 5% CO2. At two hours of incubation, brefeldin A (60ug/uL final concentration; Sigma-Aldrich) and monensin (0.7 ug/mL final concentration; BD Biosciences) were added to inhibit cytokine secretion.
T-cell stimulation panel: LIVE/DEAD Fixable Near IR (Thermo Fisher, USA), CD3-BUV395 (clone SK7), CD4-BUV737 (clone SK3), CD8-PerCPCy5.5 (clone SK1), Ki-67-FITC (clone B56), IL-2 APC (MQ1-17H12), HLA-DR (clone G46-6) from BD Biosciences, San Jose, CA. CD45RO-ECD (clone UCHL1) from Beckman Coulter (Brea, CA). CD27-BV711 (clone 0323), PD-1-BV605 (clone EH12.2H7), IFN-γ (clone B27), CD45RO-BV510 (UCHL1) from Biolegend San Diego, CA. FOXP3-PE (clone PCH101), TNF-α (clone Mab11), LIVE/DEAD Fixable Near IR from ThermoFisher Scientific, USA.

## Slide 5
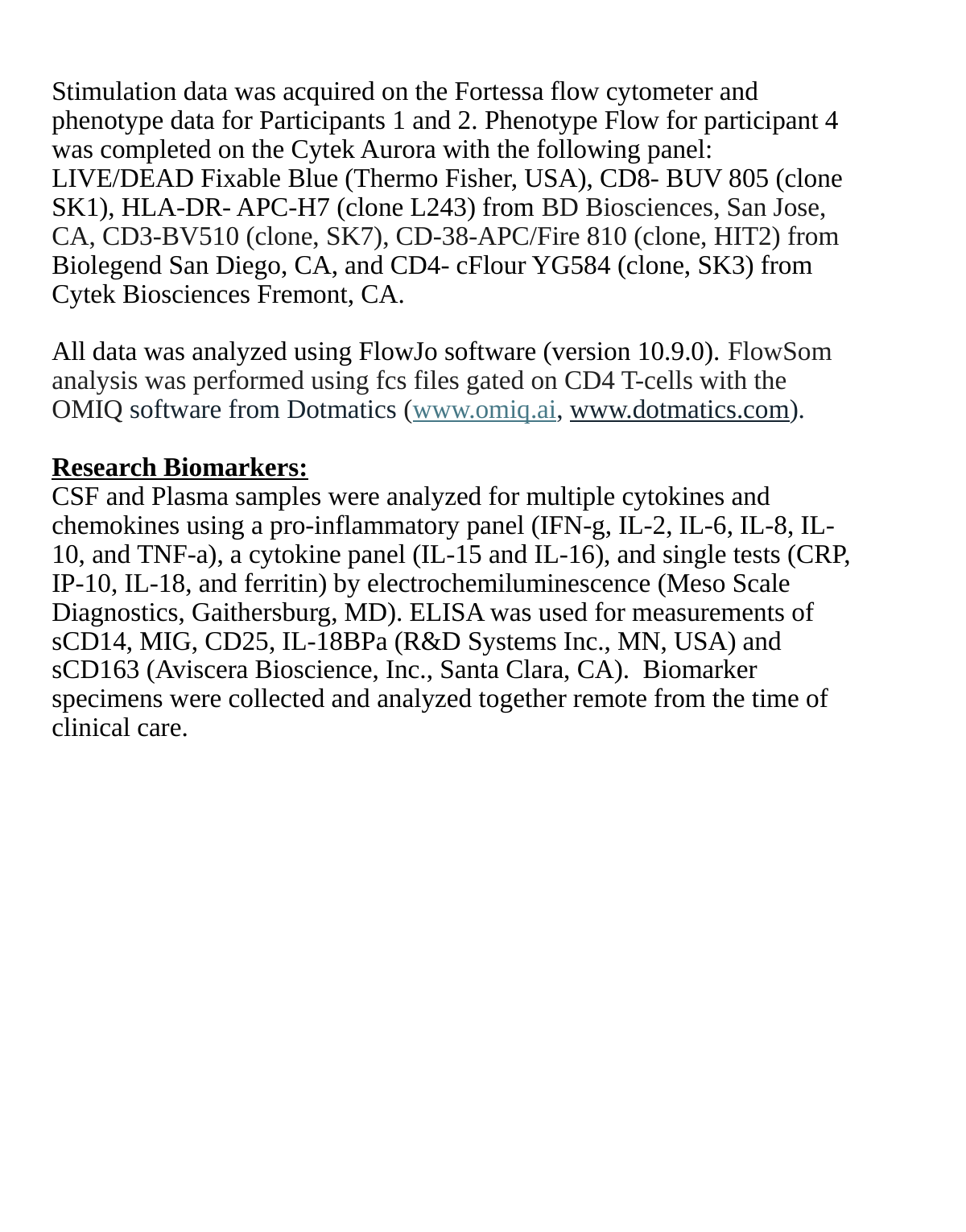

Stimulation data was acquired on the Fortessa flow cytometer and phenotype data for Participants 1 and 2. Phenotype Flow for participant 4 was completed on the Cytek Aurora with the following panel: LIVE/DEAD Fixable Blue (Thermo Fisher, USA), CD8- BUV 805 (clone SK1), HLA-DR- APC-H7 (clone L243) from BD Biosciences, San Jose, CA, CD3-BV510 (clone, SK7), CD-38-APC/Fire 810 (clone, HIT2) from Biolegend San Diego, CA, and CD4- cFlour YG584 (clone, SK3) from Cytek Biosciences Fremont, CA.
All data was analyzed using FlowJo software (version 10.9.0). FlowSom analysis was performed using fcs files gated on CD4 T-cells with the OMIQ software from Dotmatics (www.omiq.ai, www.dotmatics.com).
 
Research Biomarkers:
CSF and Plasma samples were analyzed for multiple cytokines and chemokines using a pro-inflammatory panel (IFN-g, IL-2, IL-6, IL-8, IL-10, and TNF-a), a cytokine panel (IL-15 and IL-16), and single tests (CRP, IP-10, IL-18, and ferritin) by electrochemiluminescence (Meso Scale Diagnostics, Gaithersburg, MD). ELISA was used for measurements of sCD14, MIG, CD25, IL-18BPa (R&D Systems Inc., MN, USA) and sCD163 (Aviscera Bioscience, Inc., Santa Clara, CA). Biomarker specimens were collected and analyzed together remote from the time of clinical care.

## Slide 6
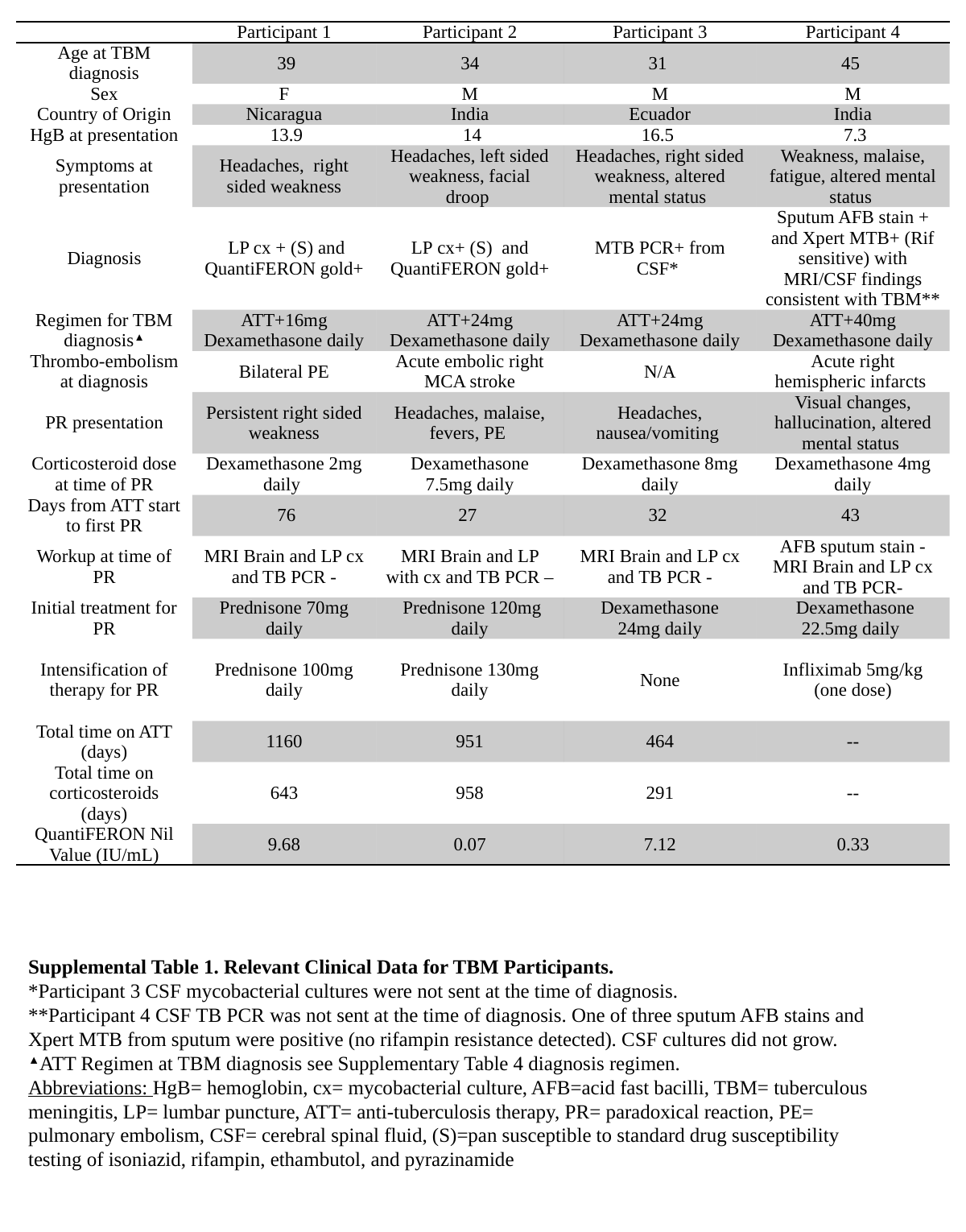

| | Participant 1 | Participant 2 | Participant 3 | Participant 4 |
| --- | --- | --- | --- | --- |
| Age at TBM diagnosis | 39 | 34 | 31 | 45 |
| Sex | F | M | M | M |
| Country of Origin | Nicaragua | India | Ecuador | India |
| HgB at presentation | 13.9 | 14 | 16.5 | 7.3 |
| Symptoms at presentation | Headaches,  right sided weakness | Headaches, left sided weakness, facial droop | Headaches, right sided weakness, altered mental status | Weakness, malaise, fatigue, altered mental status |
| Diagnosis | LP cx + (S) and QuantiFERON gold+ | LP cx+ (S)  and QuantiFERON gold+ | MTB PCR+ from CSF\* | Sputum AFB stain + and Xpert MTB+ (Rif sensitive) with MRI/CSF findings consistent with TBM\*\* |
| Regimen for TBM diagnosis▲ | ATT+16mg Dexamethasone daily | ATT+24mg Dexamethasone daily | ATT+24mg Dexamethasone daily | ATT+40mg Dexamethasone daily |
| Thrombo-embolism at diagnosis | Bilateral PE | Acute embolic right MCA stroke | N/A | Acute right hemispheric infarcts |
| PR presentation | Persistent right sided weakness | Headaches, malaise, fevers, PE | Headaches, nausea/vomiting | Visual changes, hallucination, altered mental status |
| Corticosteroid dose at time of PR | Dexamethasone 2mg daily | Dexamethasone 7.5mg daily | Dexamethasone 8mg daily | Dexamethasone 4mg daily |
| Days from ATT start to first PR | 76 | 27 | 32 | 43 |
| Workup at time of PR | MRI Brain and LP cx and TB PCR - | MRI Brain and LP with cx and TB PCR – | MRI Brain and LP cx and TB PCR - | AFB sputum stain - MRI Brain and LP cx and TB PCR- |
| Initial treatment for PR | Prednisone 70mg daily | Prednisone 120mg daily | Dexamethasone 24mg daily | Dexamethasone 22.5mg daily |
| Intensification of therapy for PR | Prednisone 100mg daily | Prednisone 130mg daily | None | Infliximab 5mg/kg (one dose) |
| Total time on ATT (days) | 1160 | 951 | 464 | -- |
| Total time on corticosteroids (days) | 643 | 958 | 291 | -- |
| QuantiFERON Nil Value (IU/mL) | 9.68 | 0.07 | 7.12 | 0.33 |
Supplemental Table 1. Relevant Clinical Data for TBM Participants.
*Participant 3 CSF mycobacterial cultures were not sent at the time of diagnosis.
**Participant 4 CSF TB PCR was not sent at the time of diagnosis. One of three sputum AFB stains and Xpert MTB from sputum were positive (no rifampin resistance detected). CSF cultures did not grow.
▲ATT Regimen at TBM diagnosis see Supplementary Table 4 diagnosis regimen.
Abbreviations: HgB= hemoglobin, cx= mycobacterial culture, AFB=acid fast bacilli, TBM= tuberculous meningitis, LP= lumbar puncture, ATT= anti-tuberculosis therapy, PR= paradoxical reaction, PE= pulmonary embolism, CSF= cerebral spinal fluid, (S)=pan susceptible to standard drug susceptibility testing of isoniazid, rifampin, ethambutol, and pyrazinamide

## Slide 7
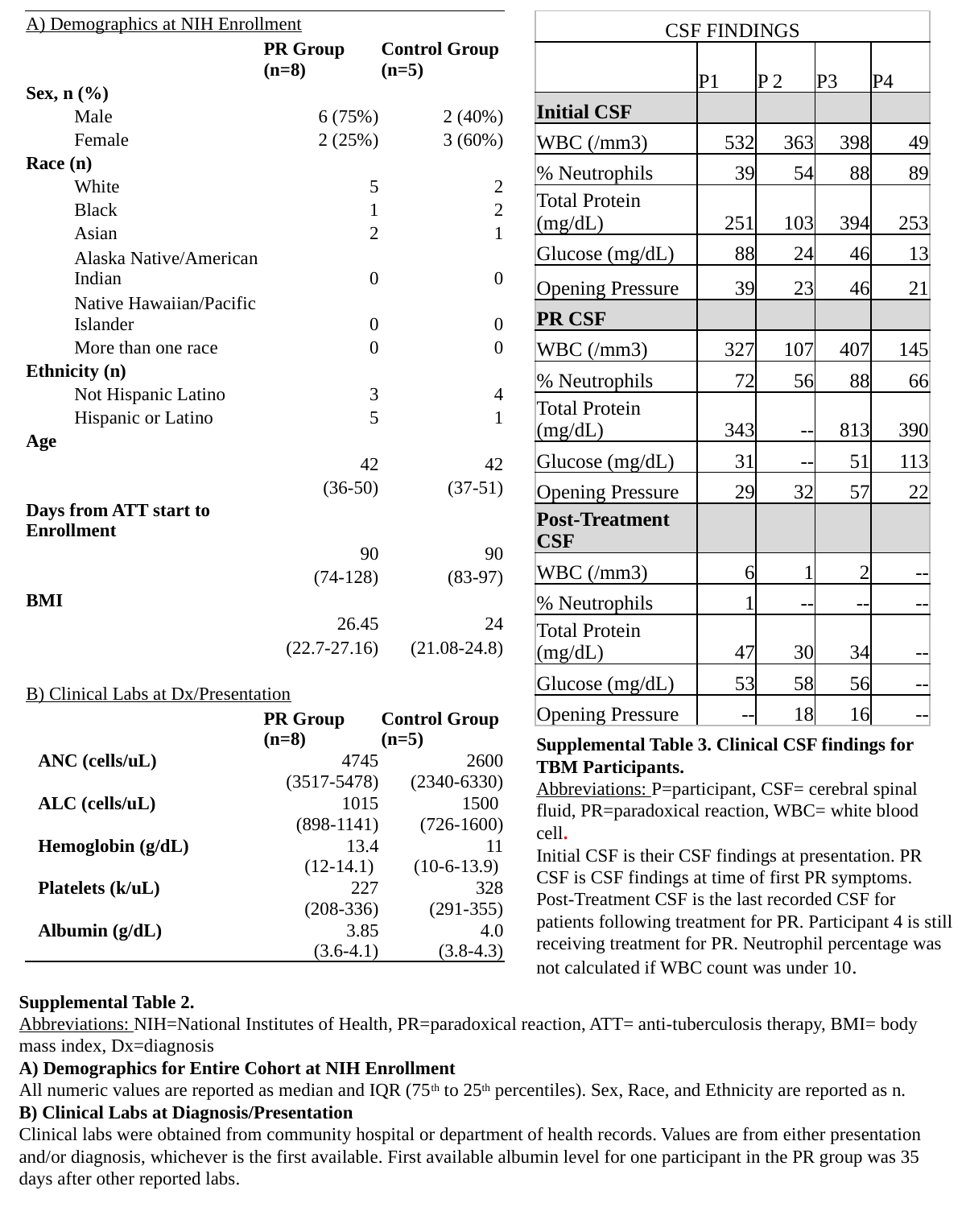

| A) Demographics at NIH Enrollment | | |
| --- | --- | --- |
| | PR Group (n=8) | Control Group (n=5) |
| Sex, n (%) | | |
| Male | 6 (75%) | 2 (40%) |
| Female | 2 (25%) | 3 (60%) |
| Race (n) | | |
| White | 5 | 2 |
| Black | 1 | 2 |
| Asian | 2 | 1 |
| Alaska Native/American Indian | 0 | 0 |
| Native Hawaiian/Pacific Islander | 0 | 0 |
| More than one race | 0 | 0 |
| Ethnicity (n) | | |
| Not Hispanic Latino | 3 | 4 |
| Hispanic or Latino | 5 | 1 |
| Age | | |
| | 42 | 42 |
| | (36-50) | (37-51) |
| Days from ATT start to Enrollment | | |
| | 90 | 90 |
| | (74-128) | (83-97) |
| BMI | | |
| | 26.45 | 24 |
| | (22.7-27.16) | (21.08-24.8) |
| B) Clinical Labs at Dx/Presentation | | |
| | PR Group (n=8) | Control Group (n=5) |
| ANC (cells/uL) | 4745 (3517-5478) | 2600 (2340-6330) |
| ALC (cells/uL) | 1015 (898-1141) | 1500 (726-1600) |
| Hemoglobin (g/dL) | 13.4 (12-14.1) | 11 (10-6-13.9) |
| Platelets (k/uL) | 227 (208-336) | 328 (291-355) |
| Albumin (g/dL) | 3.85 (3.6-4.1) | 4.0 (3.8-4.3) |
| CSF FINDINGS | | | | |
| --- | --- | --- | --- | --- |
| | P1 | P 2 | P3 | P4 |
| Initial CSF | | | | |
| WBC (/mm3) | 532 | 363 | 398 | 49 |
| % Neutrophils | 39 | 54 | 88 | 89 |
| Total Protein (mg/dL) | 251 | 103 | 394 | 253 |
| Glucose (mg/dL) | 88 | 24 | 46 | 13 |
| Opening Pressure | 39 | 23 | 46 | 21 |
| PR CSF | | | | |
| WBC (/mm3) | 327 | 107 | 407 | 145 |
| % Neutrophils | 72 | 56 | 88 | 66 |
| Total Protein (mg/dL) | 343 | -- | 813 | 390 |
| Glucose (mg/dL) | 31 | -- | 51 | 113 |
| Opening Pressure | 29 | 32 | 57 | 22 |
| Post-Treatment CSF | | | | |
| WBC (/mm3) | 6 | 1 | 2 | -- |
| % Neutrophils | 1 | -- | -- | -- |
| Total Protein (mg/dL) | 47 | 30 | 34 | -- |
| Glucose (mg/dL) | 53 | 58 | 56 | -- |
| Opening Pressure | -- | 18 | 16 | -- |
Supplemental Table 3. Clinical CSF findings for TBM Participants.
Abbreviations: P=participant, CSF= cerebral spinal fluid, PR=paradoxical reaction, WBC= white blood cell.
Initial CSF is their CSF findings at presentation. PR CSF is CSF findings at time of first PR symptoms. Post-Treatment CSF is the last recorded CSF for patients following treatment for PR. Participant 4 is still receiving treatment for PR. Neutrophil percentage was not calculated if WBC count was under 10.
Supplemental Table 2.
Abbreviations: NIH=National Institutes of Health, PR=paradoxical reaction, ATT= anti-tuberculosis therapy, BMI= body mass index, Dx=diagnosis
A) Demographics for Entire Cohort at NIH Enrollment
All numeric values are reported as median and IQR (75th to 25th percentiles). Sex, Race, and Ethnicity are reported as n.
B) Clinical Labs at Diagnosis/Presentation
Clinical labs were obtained from community hospital or department of health records. Values are from either presentation and/or diagnosis, whichever is the first available. First available albumin level for one participant in the PR group was 35 days after other reported labs.

## Slide 8
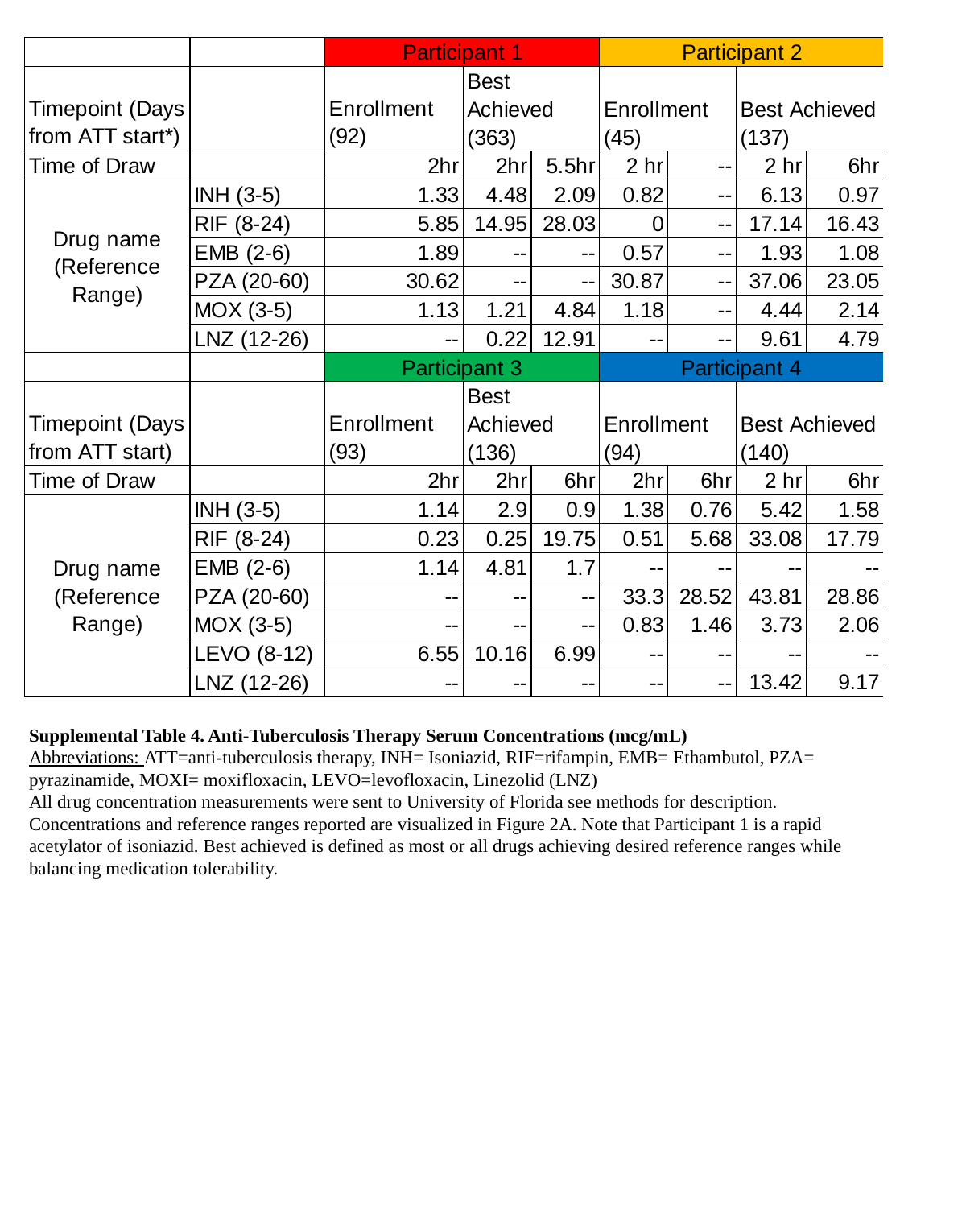

Supplemental Table 4. Anti-Tuberculosis Therapy Serum Concentrations (mcg/mL)
Abbreviations: ATT=anti-tuberculosis therapy, INH= Isoniazid, RIF=rifampin, EMB= Ethambutol, PZA= pyrazinamide, MOXI= moxifloxacin, LEVO=levofloxacin, Linezolid (LNZ)
All drug concentration measurements were sent to University of Florida see methods for description. Concentrations and reference ranges reported are visualized in Figure 2A. Note that Participant 1 is a rapid acetylator of isoniazid. Best achieved is defined as most or all drugs achieving desired reference ranges while balancing medication tolerability.

## Slide 9
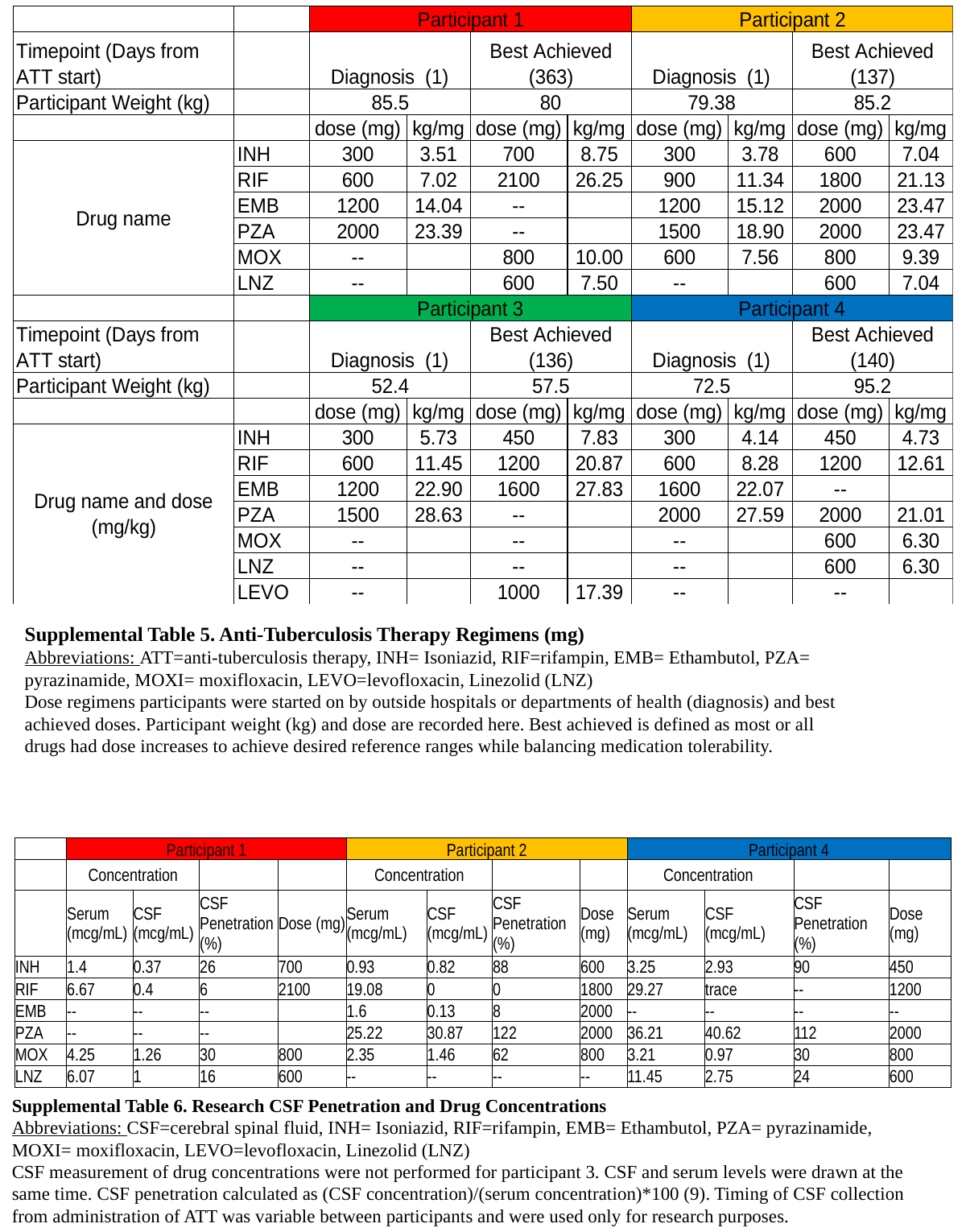

Supplemental Table 5. Anti-Tuberculosis Therapy Regimens (mg)
Abbreviations: ATT=anti-tuberculosis therapy, INH= Isoniazid, RIF=rifampin, EMB= Ethambutol, PZA= pyrazinamide, MOXI= moxifloxacin, LEVO=levofloxacin, Linezolid (LNZ)
Dose regimens participants were started on by outside hospitals or departments of health (diagnosis) and best achieved doses. Participant weight (kg) and dose are recorded here. Best achieved is defined as most or all drugs had dose increases to achieve desired reference ranges while balancing medication tolerability.
| | Participant 1 | | | | Participant 2 | | | | Participant 4 | | | |
| --- | --- | --- | --- | --- | --- | --- | --- | --- | --- | --- | --- | --- |
| | Concentration | | | | Concentration | | | | Concentration | | | |
| | Serum (mcg/mL) | CSF (mcg/mL) | CSF Penetration (%) | Dose (mg) | Serum (mcg/mL) | CSF (mcg/mL) | CSF Penetration (%) | Dose (mg) | Serum (mcg/mL) | CSF (mcg/mL) | CSF Penetration (%) | Dose (mg) |
| INH | 1.4 | 0.37 | 26 | 700 | 0.93 | 0.82 | 88 | 600 | 3.25 | 2.93 | 90 | 450 |
| RIF | 6.67 | 0.4 | 6 | 2100 | 19.08 | 0 | 0 | 1800 | 29.27 | trace | -- | 1200 |
| EMB | -- | -- | -- | | 1.6 | 0.13 | 8 | 2000 | -- | -- | -- | -- |
| PZA | -- | -- | -- | | 25.22 | 30.87 | 122 | 2000 | 36.21 | 40.62 | 112 | 2000 |
| MOX | 4.25 | 1.26 | 30 | 800 | 2.35 | 1.46 | 62 | 800 | 3.21 | 0.97 | 30 | 800 |
| LNZ | 6.07 | 1 | 16 | 600 | -- | -- | -- | -- | 11.45 | 2.75 | 24 | 600 |
Supplemental Table 6. Research CSF Penetration and Drug Concentrations
Abbreviations: CSF=cerebral spinal fluid, INH= Isoniazid, RIF=rifampin, EMB= Ethambutol, PZA= pyrazinamide, MOXI= moxifloxacin, LEVO=levofloxacin, Linezolid (LNZ)
CSF measurement of drug concentrations were not performed for participant 3. CSF and serum levels were drawn at the same time. CSF penetration calculated as (CSF concentration)/(serum concentration)*100 (9). Timing of CSF collection from administration of ATT was variable between participants and were used only for research purposes.

## Slide 10
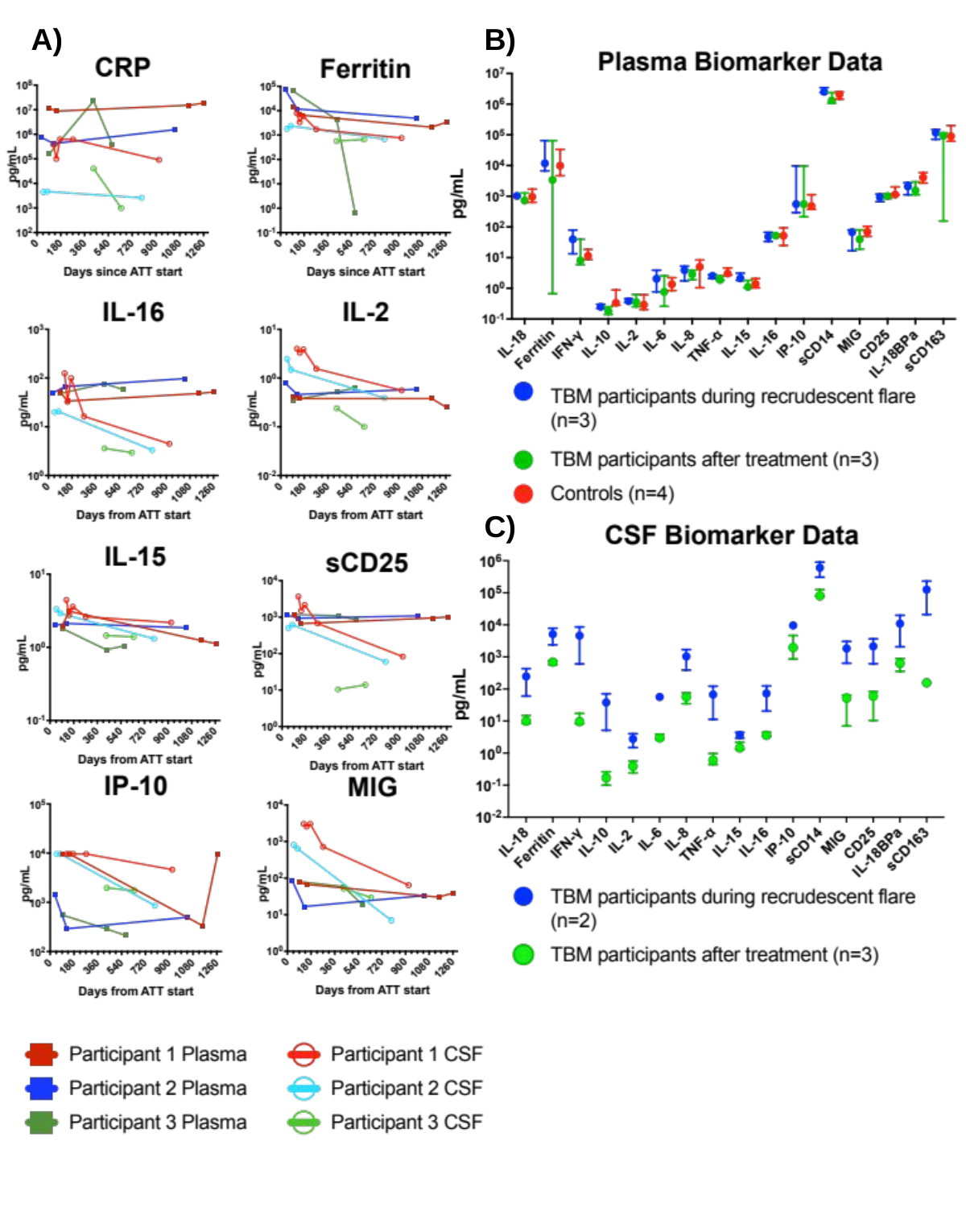

A)
B)
C)

## Slide 11
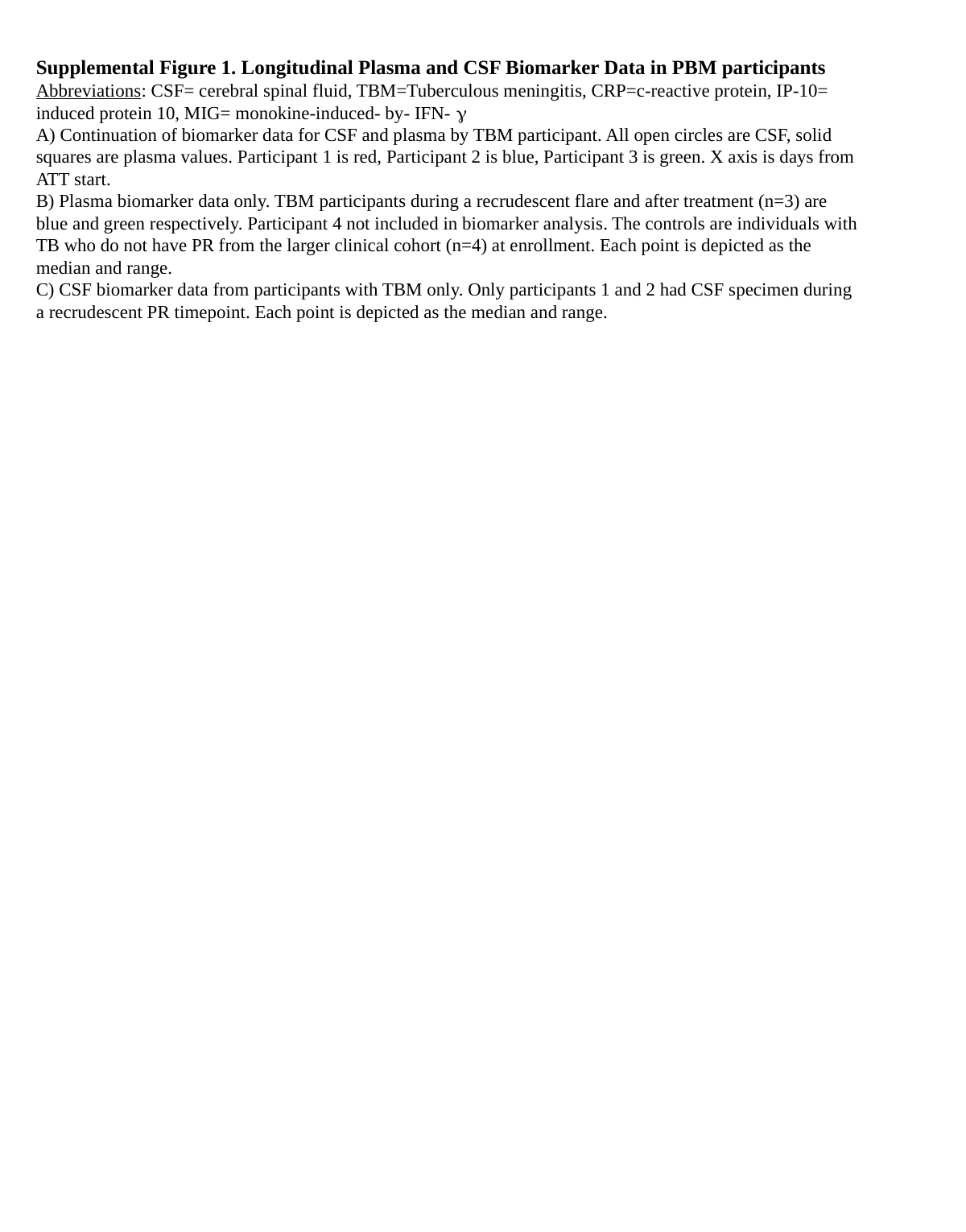

Supplemental Figure 1. Longitudinal Plasma and CSF Biomarker Data in PBM participants
Abbreviations: CSF= cerebral spinal fluid, TBM=Tuberculous meningitis, CRP=c-reactive protein, IP-10= induced protein 10, MIG= monokine-induced- by- IFN- γ
A) Continuation of biomarker data for CSF and plasma by TBM participant. All open circles are CSF, solid squares are plasma values. Participant 1 is red, Participant 2 is blue, Participant 3 is green. X axis is days from ATT start.
B) Plasma biomarker data only. TBM participants during a recrudescent flare and after treatment (n=3) are blue and green respectively. Participant 4 not included in biomarker analysis. The controls are individuals with TB who do not have PR from the larger clinical cohort (n=4) at enrollment. Each point is depicted as the median and range.
C) CSF biomarker data from participants with TBM only. Only participants 1 and 2 had CSF specimen during a recrudescent PR timepoint. Each point is depicted as the median and range.

## Slide 12
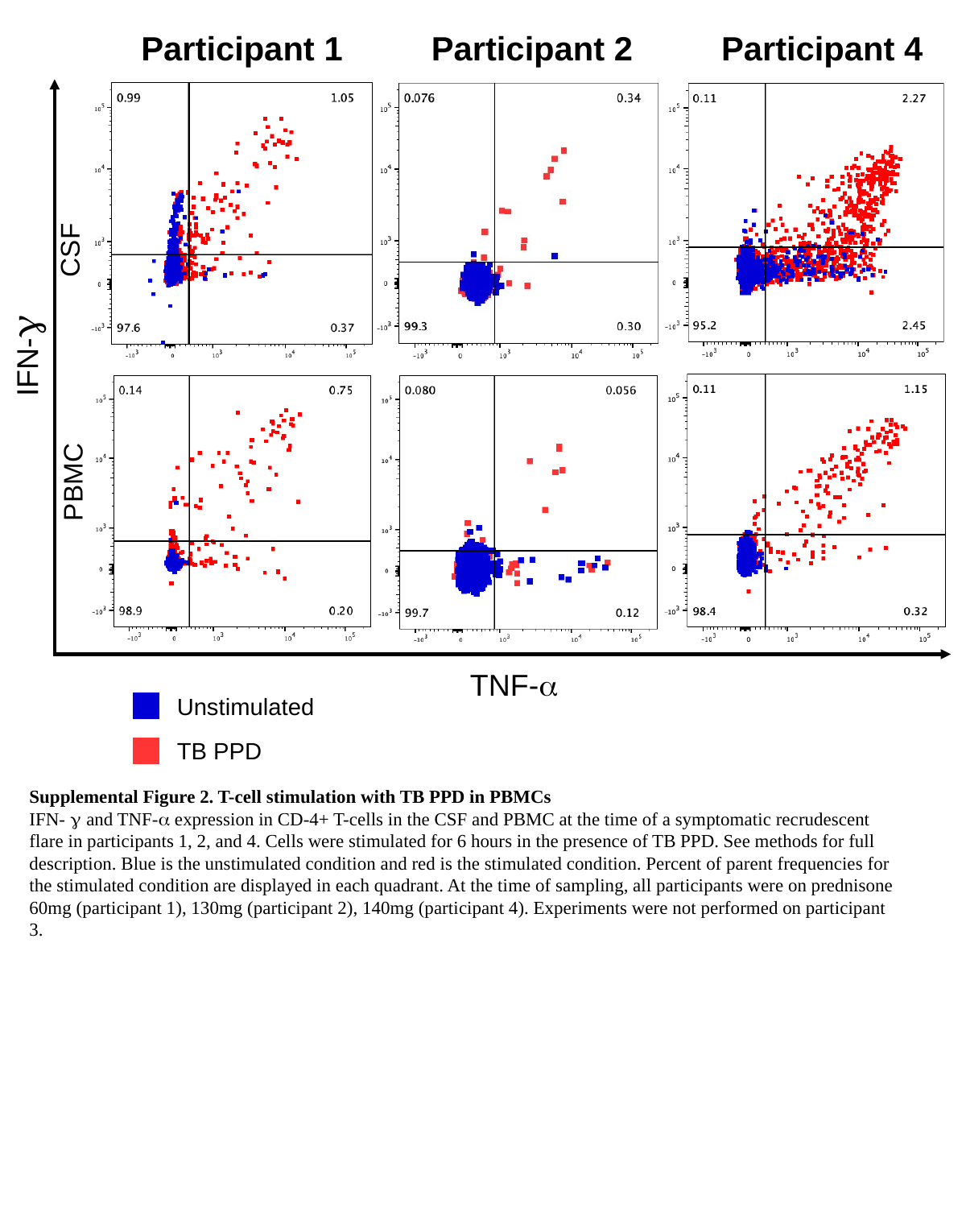

Participant 4
Participant 1
Participant 2
CSF
IFN-γ
PBMC
TNF-α
Unstimulated
TB PPD
Supplemental Figure 2. T-cell stimulation with TB PPD in PBMCs
IFN- γ and TNF-α expression in CD-4+ T-cells in the CSF and PBMC at the time of a symptomatic recrudescent flare in participants 1, 2, and 4. Cells were stimulated for 6 hours in the presence of TB PPD. See methods for full description. Blue is the unstimulated condition and red is the stimulated condition. Percent of parent frequencies for the stimulated condition are displayed in each quadrant. At the time of sampling, all participants were on prednisone 60mg (participant 1), 130mg (participant 2), 140mg (participant 4). Experiments were not performed on participant 3.

## Slide 13
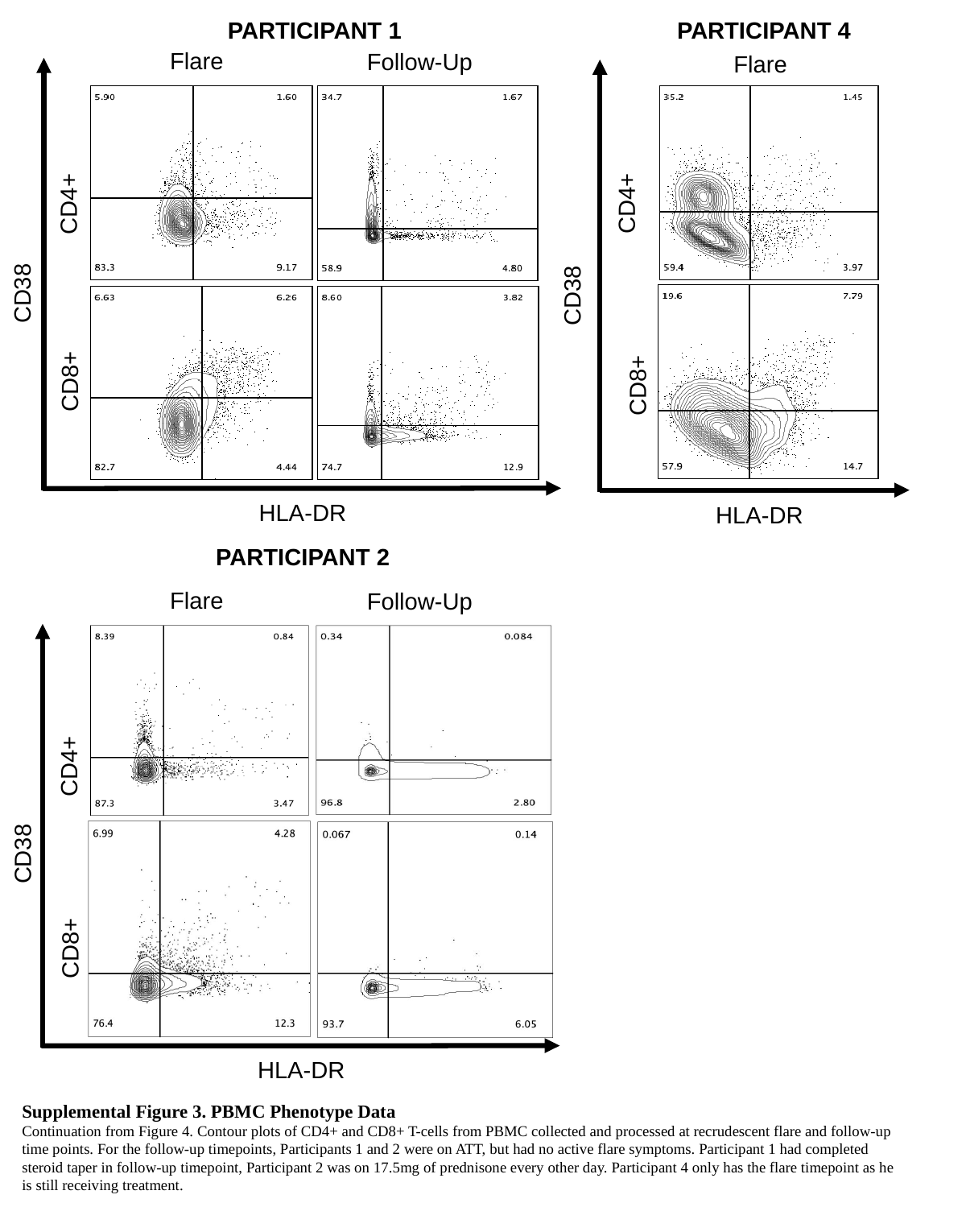

PARTICIPANT 1
PARTICIPANT 4
Flare
Follow-Up
Flare
CD4+
CD4+
CD38
CD38
CD8+
CD8+
HLA-DR
HLA-DR
PARTICIPANT 2
Flare
Follow-Up
CD4+
CD38
CD8+
HLA-DR
Supplemental Figure 3. PBMC Phenotype Data
Continuation from Figure 4. Contour plots of CD4+ and CD8+ T-cells from PBMC collected and processed at recrudescent flare and follow-up time points. For the follow-up timepoints, Participants 1 and 2 were on ATT, but had no active flare symptoms. Participant 1 had completed steroid taper in follow-up timepoint, Participant 2 was on 17.5mg of prednisone every other day. Participant 4 only has the flare timepoint as he is still receiving treatment.
